# Analysis of alpha‐1‐antitrypsin (AAT)‐regulated, glucocorticoid receptor‐dependent genes in macrophages reveals a novel host defense function of AAT

**DOI:** 10.14814/phy2.16124

**Published:** 2024-07-17

**Authors:** Xiyuan Bai, Junfeng Gao, Xiaoyu Guan, Drew E. Narum, Lorelenn B. Fornis, David E. Griffith, Bifeng Gao, Robert A. Sandhaus, Hua Huang, Edward D. Chan

**Affiliations:** ^1^ Department of Medicine Rocky Mountain Regional Veterans Affairs Medical Center Aurora Colorado USA; ^2^ Department of Academic Affairs National Jewish Health Denver Colorado USA; ^3^ Division of Pulmonary Sciences and Critical Care Medicine University of Colorado Anschutz Medical Campus Aurora Colorado USA; ^4^ Department of Immunology and Genomic Medicine National Jewish Health Denver Colorado USA; ^5^ Department of Biostatistics and Informatics University of Colorado School of Public Health Anschutz Medical Campus Aurora Colorado USA; ^6^ Department of Medicine National Jewish Health Denver Colorado USA; ^7^ Department of Immunology and Microbiology University of Colorado School of Medicine Aurora Colorado USA

**Keywords:** AAT, gene regulation, glucocorticoid receptor, GM‐CSF, serine protease

## Abstract

Alpha‐1‐antitrypsin (AAT) plays a homeostatic role in attenuating excessive inflammation and augmenting host defense against microbes. We demonstrated previously that AAT binds to the glucocorticoid receptor (GR) resulting in significant anti‐inflammatory and antimycobacterial consequences in macrophages. Our current investigation aims to uncover AAT‐regulated genes that rely on GR in macrophages. We incubated control THP‐1 cells (THP‐1^control^) and THP‐1 cells knocked down for GR (THP‐1^GR‐KD^) with AAT, performed bulk RNA sequencing, and analyzed the findings. In THP‐1^control^ cells, AAT significantly upregulated 408 genes and downregulated 376 genes. Comparing THP‐1^control^ and THP‐1^GR‐KD^ cells, 125 (30.6%) of the AAT‐upregulated genes and 154 (41.0%) of the AAT‐downregulated genes were significantly dependent on GR. Among the AAT‐upregulated, GR‐dependent genes, *CSF‐2* that encodes for granulocyte‐monocyte colony‐stimulating factor (GM‐CSF), known to be host‐protective against nontuberculous mycobacteria, was strongly upregulated by AAT and dependent on GR. We further quantified the mRNA and protein of several AAT‐upregulated, GR‐dependent genes in macrophages and the mRNA of several AAT‐downregulated, GR‐dependent genes. We also discussed the function(s) of selected AAT‐regulated, GR‐dependent gene products largely in the context of mycobacterial infections. In conclusion, AAT regulated several genes that are dependent on GR and play roles in host immunity against mycobacteria.

## Graphical abstract

Alpha‐1‐antitrypsin (AAT) binds to the glucocorticoid receptor (GR) with anti‐inflammatory and antimycobacterial consequences in macrophages. AAT significantly induced expression of 408 genes, and 31% of the induced genes were dependent on GR; AAT inhibited expression of 376 genes, and 41% of the inhibited genes were dependent on GR. Colony‐stimulating factor‐2 (CSF‐2) gene that encodes for granulocyte‐monocyte colony‐stimulating factor (GM‐CSF) and is known to be host‐protective against mycobacteria, was strongly induced by AAT and dependent on GR.

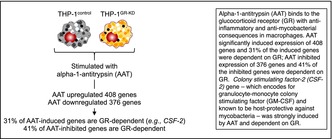

## 1 INTRODUCTION

Alpha‐1‐antitrypsin (AAT), the most abundant serine protease inhibitor (serpin) in circulation, is produced and secreted mainly by hepatocytes, but also by other cell types, including intestinal cells, lung alveolar cells, neutrophils, and macrophages (Janciauskiene et al., 2018; Pini et al., 2014). The best‐known function of AAT is the irreversible inhibition of elastase, but AAT also binds and inactivates other serine proteases such as proteinase‐3, trypsin, chymotrypsin, myeloperoxidase, cathepsins, α‐defensins, tryptase, plasmin, thrombin, factor Xa, urokinase, ADAM17 (a disintegrin and metalloprotease 17, aka tumor necrosis factor converting enzyme), and Transmembrane Protease 2 (TMPRSS2) (Bai et al., 2023; Bai, Buckle, et al., 2022; Frenzel et al., 2015; O'Brien et al., 2022). AAT has other biological functions that are less well appreciated; among these are anti‐inflammatory and host defense properties (Bai et al., 2019; Bai, Bai, et al., 2022; Jonigk et al., 2013; Lewis, 2012; Wanner et al., 2012). For example, we previously showed that AAT reduces the burden in *Mycobacterium intracellulare* in primary human monocyte‐derived macrophages via sequential inhibition of both nuclear factor‐kappa B (NFκB) and A20 (a deubiquitinating enzyme that normally inhibits autophagosome maturation), thus inducing autophagy, a known killing mechanism of intracellular mycobacteria (Bai et al., 2019). The mechanisms by which AAT affects these noncanonical functions are varied and incompletely understood but likely involve gene regulation. While a receptor for AAT has not been described, AAT is known to enter cells through endocytosis by low‐density lipoprotein (LDL) receptor related protein‐1 (LRP1) or through clathrin‐coated vesicles and caveolae‐mediated mechanisms (Serban & Petrache, 2016; Sohrab et al., 2009; Zhou et al., 2015).

The glucocorticoid receptor (GR) resides in the cytoplasm, and upon binding to its canonical ligand—endogenous and exogenous glucocorticoids—the glucocorticoid GR complex enhances or inhibits the expression of numerous genes involved in an array of cellular processes including cytokine signaling and apoptosis (Cain & Cidlowski, 2017; Necela & Cidlowski, 2004; Oakley & Cidlowski, 2013). We recently reported that AAT binds to the GR and that the AAT–GR complex inhibited lipopolysaccharide‐induced NFκB activation and interleukin‐8 (IL‐8) production as well as reduced the burden of *Mycobacterium tuberculosis* and *M. intracellulare* in macrophages (Bai, Bai, et al., 2022). These biological effects of AAT–GR indicate that AAT likely regulates gene expression, but a more detailed analysis of AAT‐regulated genes that are dependent on GR has not been reported.

To determine the repertoire of AAT‐regulated, GR‐dependent genes in macrophages, we performed bulk RNA sequencing (RNA‐seq) of control THP‐1 cells (THP‐1^control^) and THP‐1 cells stably knocked down for GR (THP‐1^GR‐KD^) following stimulation of both cell populations with AAT. We found that AAT significantly induced or inhibited 5–6 percent of the ~7000 genes analyzed in macrophages and that 30%–40% of these AAT‐regulated genes were significantly dependent on GR. Since our interest in AAT lies in the context of nontuberculous mycobacterial (NTM) infections, we corroborated the bulk RNA‐seq data by quantifying the transcripts and proteins of selected AAT‐regulated genes relevant in host defense or host vulnerability to mycobacterial infections.

## 2 MATERIALS AND METHODS

### 2.1 Materials

The human monocytic cell line (THP‐1) was obtained from the American Type Culture Collection (Manassas, Virginia). Phorbol myristate acetate (PMA) was purchased from Sigma (St. Louis, MO). Fetal bovine serum (FBS) was purchased from Atlanta Biologicals (Lawrenceville, GA) and heat‐inactivated at 56°C for 30 minutes. An aliquot of the glucocorticoid receptor (*NR3C1*) human shRNA (shRNA‐GR) lentiviral particle was a kind gift from Miles Pufall, Ph.D. (University of Iowa Carver College of Medicine). RPMI‐1640 and ELISA kits for human granulocyte monocyte‐colony‐stimulating factor (GM‐CSF) (Catalog# 5018230), interleukin‐1‐beta (IL‐1β) (#5018066), and IL‐27 (#50246661) were purchased from ThermoFisher Scientific (Carlsbad, CA). RNeasy Plus Mini kit for the purification of total RNA from cells was purchased from QIAGEN (Redwood City. CA). AAT (Glassia®) (NDC 0944‐2884‐01) was acquired from Kamada Ltd., Israel. The RT‐qPCR primers were synthesized from Integrated DNA Technologies (Coralville, IA). The cDNA was synthesized using M‐MLV Reverse Transcriptase kit from Promega (Fitchburg, WI). SYBR™ Green PCR Master MIX was obtained from Applied Biosystems (ThermoFisher Scientific, #4309155).

### 2.2 Differentiated macrophage culture and stable knockdown of the glucocorticoid receptor (GR) in THP‐1 cells

Human THP‐1 cells were cultured in RPMI‐1640 medium containing 2 mM L‐glutamine (Gibco; Grand Island, NY), 10% FBS, penicillin (100 U/mL), and streptomycin (100 μg/mL) at 37°C and 5% CO_2_. THP‐1 cells were differentiated into macrophages following incubation with 15 ng/mL PMA for 24 h. We employed shRNA‐lentivirus technology to develop a pool of THP‐1 cells stably knocked down (KD) for GR (THP‐1^GR‐KD^) and control THP‐1 cells (THP‐1^control^) using shRNA‐GR‐lentivirus and shRNA‐scrambled‐lentivirus, respectively, as previously described (Bai, Bai, et al., 2022).

### 2.3 RNA isolation and RNA sequencing (RNA‐seq)

After confirming the depletion of GR mRNA and protein through RNAseq‐ and immune‐blotting, respectively, of THP‐1^GR‐KD^ cells (Bai, Bai, et al., 2022), both differentiated THP‐1^control^ and THP‐1^GR‐KD^ were incubated in medium alone or with AAT (3 mg/mL) for 48 h and then high‐quality total RNA (260 nm/280 nm ~ 2) isolated using RNeasy Plus kit according to manufacturer's instruction. The RNA‐seq libraries prepared from independent experiments were prepared as described (Li et al., 2021). Total RNA from two independent experiments was sequenced using the Illumina NovaSeq 6000 from Genomics Shared Resource at the University of Colorado Cancer Center.

### 2.4 RNA‐seq data analysis

The RNA‐seq data were analyzed as described previously (Li et al., 2021). Briefly, the raw reads (average 20 million paired end reads, two biological replicates for each treatment) were analyzed and quality checked by FastQC. The reads were aligned to the hg38 reference genome using the Spliced Transcripts Alignment to a Reference (STAR, version 2.4.0.1) software. Reads (FPKM) were assembled into reference transcripts and counted using Cufflinks (version 2.2.1). The average reads from two biological samples were calculated using Cuffmerge (version 1.0.0). The differential gene expression between the resting and stimulated samples was analyzed using Cuffdiff (version 2.2.1). Gene Ontology (GO) enrichment analysis was performed on AAT‐regulated genes by using the Database for Annotation, Visualization and Integrated Discovery (DAVID, version 6.8) (Huang et al., 2009a,b). The genes with a fold change ≥2—whether it is the ratio of AAT‐stimulated/unstimulated in THP‐1^control^ cells or of AAT‐stimulated THP‐1^GR‐KD^ /AAT‐stimulated THP‐1^control^—are classified as “highly upregulated.” The genes with a fold change ≤0.5—with the same aforementioned conditions—are classified as “highly downregulated.” Thus, AAT‐stimulated THP‐1^GR‐KD^/AAT‐stimulated THP‐1^control^ ≤0.5 would be AAT‐stimulated genes that are dependent on GR. Genes with fold changes that are >1 but <2 (or >0.5 but <1) are classified as “less changed.” Several genes in each enriched categories were selected for heatmap representations, which were generated using Expander software (version 7.2) (Ulitsky et al., 2010).

### 2.5 Reverse transcription and quantitative polymerase chain reaction (RT‐qPCR)

Total RNA isolated from differentiated THP‐1^control^ or THP‐1^GR‐KD^ macrophages‐treated with or without AAT (3 mg/mL) for 48 h. The cDNA was then prepared, and quantitative PCR was performed in a QuantStudio 7 Flex Real‐Time PCR System using SYBR™ Green qPCR Master MIX. Relative amount of mRNA = 2^[Ct(Sample)‐Ct(HPRT)]^ where HPRT is the housekeeping gene that encodes for hypoxanthine phosphoribosyltransferase 1. The specific primers are shown in Table S1.

### 2.6 ELISA

After being incubated with medium alone or with AAT (3 mg/mL) for 48 h, the supernatants of differentiated THP‐1^control^ or THP‐1^GR‐KD^ macrophages were quantified by ELISA for GM‐CSF, IL‐1β, and IL‐27 following manufacturer's instruction.

### 2.7 Statistical analysis

Replicate RNA‐seq experiments are independent and presented as mean ± SD. The ELISA data are reported as mean ± SEM of duplicate samples of three independent experiments. Group means were compared by repeated‐measures ANOVA using Fisher's least significant test or by two‐way ANOVA with Bonferroni's post hoc test. Data were graphed in Prism 9®, and comparisons were considered significant when *p* < 0.05.

## 3 RESULTS

### 3.1 Heat‐map representation of GR‐dependent genes upregulated or downregulated by AAT

**FIGURE 1:**
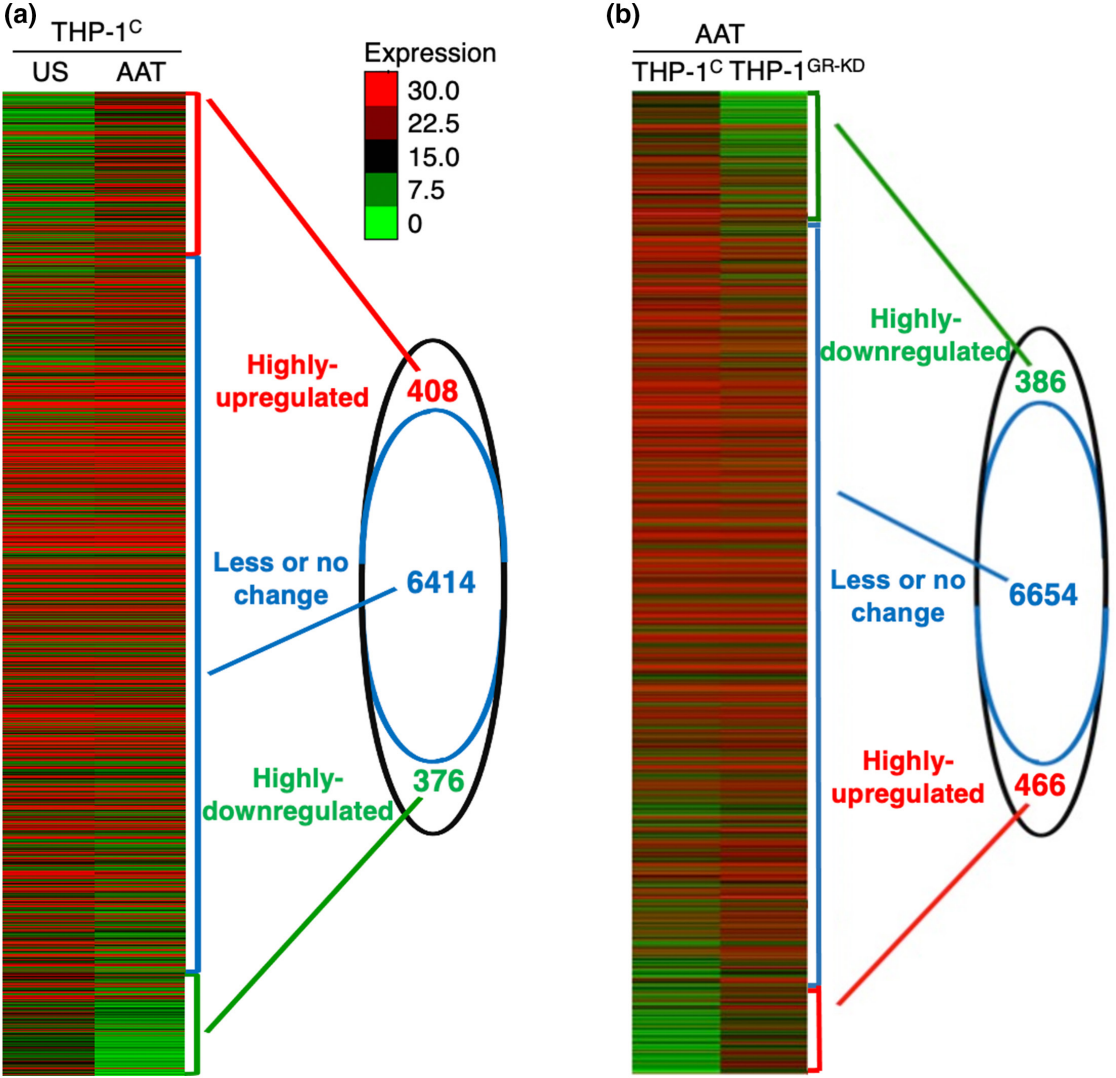
Heatmap of all the genes regulated by AAT in THP‐1 macrophages that are dependent on glucocorticoid receptor. (a) Heatmap presentation of the RNA transcripts of THP‐1^C^ macrophages incubated for 48 h in medium alone or in medium containing 3 mg/mL AAT. (b) Heatmap presentation of the RNA transcripts of THP‐1^control^ with AAT treatment compared to THP‐1^GR‐KD^. Thus, in both (a) and (b), “highly upregulated” denotes a change of ≥2‐fold, “less changed” means a change of >0.5‐fold and <2‐fold, and “highly downregulated” signifies a change of ≤0.5‐fold. Data represent duplicate of two biological samples. AAT, alpha‐1‐antitrypsin; GR, glucocorticoid receptor; THP‐1^C^, control THP‐1 cells; THP‐1^GR‐KD^, THP‐1 cells knocked down for GR; US, unstimulated.

To determine the repertoire of AAT‐regulated, GR‐dependent genes in human macrophages, THP‐1^control^ and THP‐1^GR‐KD^ cells were left unstimulated or stimulated with 3 mg/mL AAT for 48 h and total RNA isolated and sequenced. Differential expression analysis revealed that of the 7198 genes analyzed, 408 (5.7%) genes were highly upregulated (fold change ≥2) by AAT, while 376 (5.2%) genes were highly downregulated (fold change ≤0.5) by AAT. The remaining 6414 genes (89.1%) exhibited less changes (falling within fold changes of >0.5 and <1 or >1 and <2) or no change (fold change 1) (Figure 1a). There were 386 AAT‐upregulated genes that significantly decreased following GR knockdown (fold change ≤0.5 for AAT‐stimulated THP‐1^GR‐KD^/AAT‐stimulated THP‐1^control^), whereas 466 genes demonstrated significant upregulation following GR knockdown (fold change ≥2 for AAT‐stimulated THP‐1^GR‐KD^ /AAT‐stimulated THP‐1^control^) (Figure 1b). Among the 408 genes significantly upregulated by AAT (Figure 1a), 125 genes (30.6%) were variably and significantly dependent on GR (fold change ≤0.5) (Table S2). Similarly, of the 376 genes significantly downregulated by AAT (Figure 1a), 154 (41.0%) were variably and significantly dependent on GR (fold change ≥2) (Table S3).

### 3.2 AAT upregulated or downregulated a wide array of genes in macrophages

**FIGURE 2:**
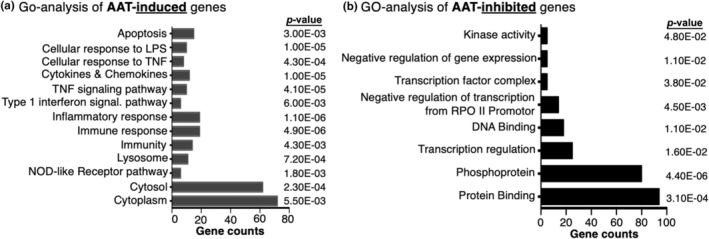
Gene Ontology (GO) enrichment analysis of AAT‐regulated genes using DAVID, version 6.8. (a) AAT‐*induced* genes sorted by various functional and subcellular categories. (b) AAT‐*inhibited* genes sorted by various functional and subcellular categories. *p*‐Values were calculated by a one‐side Fisher's exact test with the adjustment of Benjamini–Hochberg Method. Data represent duplicate of two biological samples. AAT, alpha‐1‐antitrypsin; LPS, lipopolysaccharide; RPO II, RNA polymerase II; TNF, tumor necrosis factor.

**FIGURE 3:**
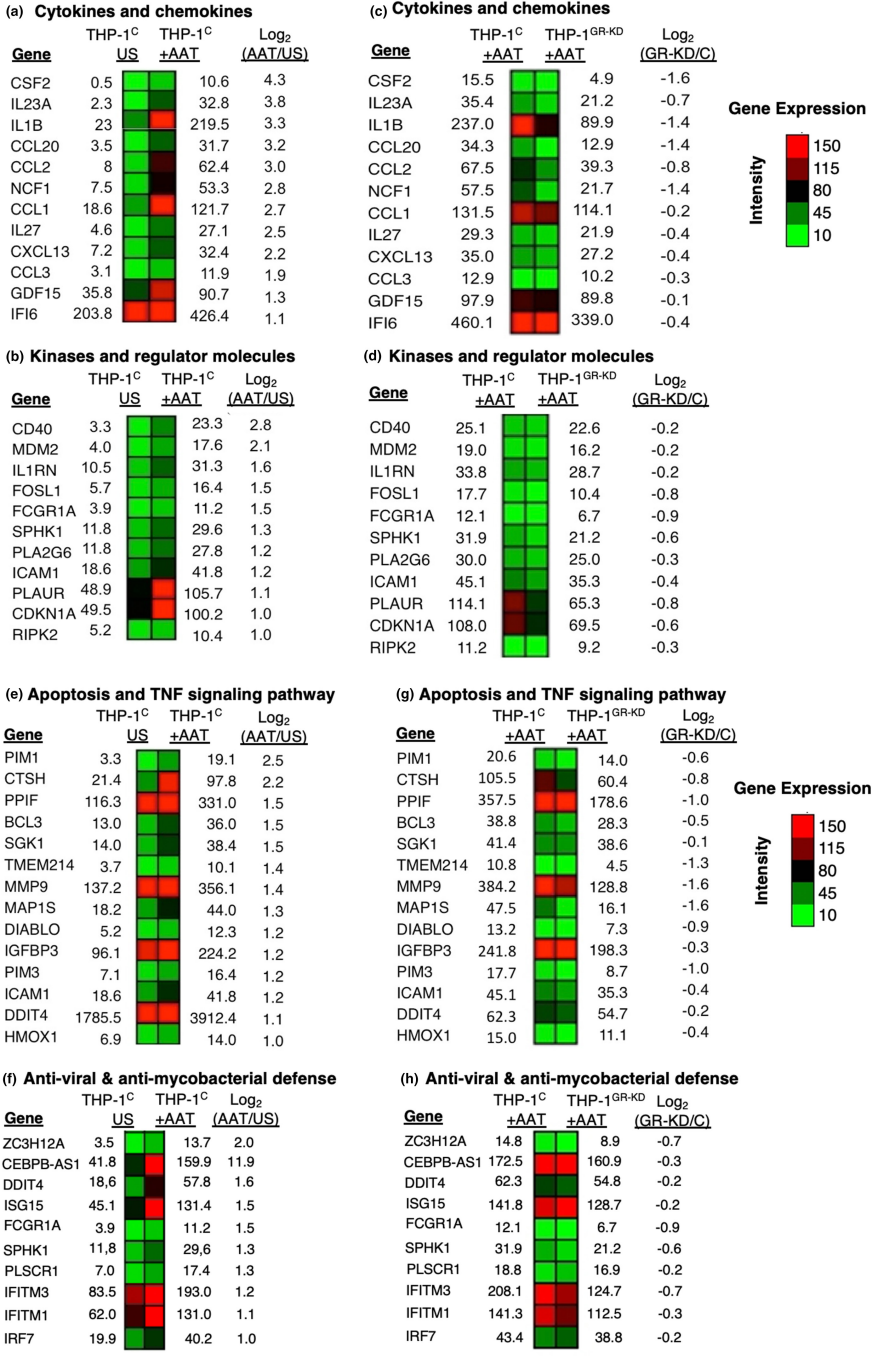
Heatmap of selected AAT‐induced, GR‐dependent genes. AAT‐induced gene expression with genes sorted by (a) cytokines and chemokines, (b) kinase and regulator molecules, (e) apoptosis and TNF signaling pathway, and (f) antiviral and antimycobacterial defense categories. (c, d, g, h) AAT‐induced, GR‐dependent gene expression sorted by the same categories, respectively. The numbers indicate Reads per Kilobase Million (RPKM). (a–h) represent duplicate of two biological samples. AAT, alpha‐1‐antitrypsin; C, control THP‐1 cells; GR‐KD, THP‐1 cells knocked down for glucocorticoid receptor; THP‐1^C^, control THP‐1 cells; THP‐1^GR‐KD^, THP‐1 cells knocked down for the glucocorticoid receptor; US, unstimulated.

Gene‐ontology (GO) enrichment analysis was used to categorize AAT‐upregulated genes (Figure 2a) and AAT‐downregulated genes (Figure 2b) into specific pathways. We then categorized specific AAT‐upregulated, GR‐dependent genes into four pathways: (i) cytokines and chemokines (Figure 3a,c); (ii) kinases and regulator molecules (Figure 3b,d); (iii) apoptosis and tumor necrosis factor (TNF) signaling (Figure 3e,g); and (iv) antiviral and antimycobacterial defense (Figure 3f,h). A brief description of some of the AAT‐induced, GR‐dependent genes and gene products (shown in Figure 3a–h) that are relevant for mycobacterial infections is shown in Table 1. Brief discussions on the function of additional selected gene products are shown in Tables S4–S9. As seen in Table 1, the mycobacteria‐relevant genes that are AAT‐upregulated and GR‐dependent may be categorized into a growth factor (*CSF2*), cytokines (*IL‐1B*, *IL23A*, *IL27B*), chemokines (*CCL1*, *CCL2*, *CCL3*, *CCL20*), and other effector proteins (*IFI6* and *NCF1*). *CSF2* that encodes for granulocyte‐monocyte colony‐stimulating factor (GM‐CSF) is strongly upregulated by AAT (by 20‐fold) (Figure 3a).

**TABLE 1:** AAT‐upregulated, GR‐dependent genes relevant for mycobacterial infections.

Gene	Protein	Brief synopsis of function
*CSF2*	GM‐CSF	See Section 4
*IL1B*	Interleukin‐1β (IL‐1β)	IL‐1β, an essential component of inflammasomes, is host‐protective against various pathogens (Lopez‐Castejon & Brough, 2011; Rastogi & Briken, 2022). Compared with PBMC from healthy household controls, stimulated PBMC of NTM lung disease subjects had reduced production of IL‐1 family of cytokines (IL‐1α, IL‐1β, and IL‐18) (Jung et al., 2022).
*IL23A*	Interleukin 23 alpha subunit (p19)	IL‐23 is essential for differentiation of undifferentiated T cells to the T_H_1 and T_H_17 phenotypes (Lee et al., 2017) as well as to IFNγ‐dependent in Vδ2^+^ γδ T cells and MAIT cells (Ogishi et al., 2022; Philippot et al., 2023), all important in host immunity against mycobacteria.
*IL27B*	IL‐27 beta subunit (EBI3)	IL‐27, a heterodimeric cytokine, regulates both innate and adaptive immunity via Jak–Stat signaling. IL‐27 induces IFNγ and inflammatory mediators from innate immune cells and T cells and plays important role in the host immunity against *Mycobacterium tuberculosis* (*MTB*) (Abdalla et al., 2015).
*CCL1*	C‐C motif chemokine ligand 1 (CCL1), aka I‐309, binds receptor CCR8.	CCL1 is induced by *MTB* and TLR ligands in macrophages (Zhao et al., 2015). CCL1 induces the migration of human monocytes (Miller & Krangel, 1992) and mediates T_H_2 cell and T regulatory cell trafficking (Griffith et al., 2014). CCL1 as part of a four‐biomarker signature (along with RANTES, CRP, and MIP‐1α) was relatively specific in distinguishing active TB from latent TB (Chendi et al., 2021; Wei et al., 2015). CCL1 polymorphisms may be associated with increased susceptibility to certain forms of TB (Thuong et al., 2008).
*CCL2*	C‐C motif chemokine ligand 2, aka monocyte chemoattractant protein‐1 (MCP‐1), binds receptor CCR2.	CCL2 is essential for recruitment of monocytes and T cells. Increased expression of CCL2 with pulmonary TB (as compared to extrapulmonary TB) may help keep TB localized to the lungs (Hasan et al., 2005). In mice, overexpression of CCL2 in the lungs protects against *M. bovis* BCG (Schreiber et al., 2008). CCL2 may be induced by either T_H_1 or T_H_2 cytokines, thus participating in both type 1 and type 2 granulomas (Chiu et al., 2003). Increased levels in plasma is a potential indicator of TB severity (Domingo‐Gonzalez et al., 2016). CCL2 may increase HIV pathogenesis, which could be detrimental in HIV‐TB coinfection (Ansari et al., 2013).
*CCL3*	C‐C motif chemokine ligand 3 (CCL3), aka macrophage inflammatory protein 1‐alpha (MIPα), binds to receptor CCR5.	Chemoattractant for neutrophils, monocytes, and macrophages. All CCR5 ligands (CCL3, CCL4, & CCL5) are upregulated in the lungs of a murine model of *MTB* infection (Domingo‐Gonzalez et al., 2016). Yet, the CCR5 knockout mice recruit adequate number of lymphocytes to the lungs and were not compromised in controlling *MTB* infection (Algood & Flynn, 2004). Infection of mice with the rough strain of *M. avium* (compared to the smooth strain) resulted in more severe disease and greater production of pro‐inflammatory cytokines, CCL3, and CCL5 (Nishimura et al., 2020).
*CCL20*	C‐C motif chemokine ligand 20, aka macrophage‐inflammatory protein‐3α (MIP‐3α)	CCL20 is a chemokine for lymphocytes, especially T_H_17 and T regulatory cells and immature dendritic cells. CCL20 has been shown to have direct antimicrobial effects against a wide variety of pyogenic bacteria (Yang et al., 2003). Compared to NTM, *MTB* induces higher levels CCL20 protein expression (Rivero‐Lezcano et al., 2010). CCL20 also attenuates mycobacteria‐induced reactive O_2_ species and apoptosis (Rivero‐Lezcano et al., 2010).
*IFI6*	Interferon alpha inducible protein 6 (IFI‐6)	IFI‐6 negatively regulates innate immunity by interacting with retinoic acid‐inducible gene I (RIG‐I), reducing interferons (type I IFNs), IFN‐stimulated genes, and pro‐inflammatory cytokines (Villamayor et al., 2023). *MTB* also induces IFI‐6 in human macrophages (Zhou et al., 2019).
*NCF1*	Neutrophil cytosolic factor 1	*NCF1* encodes a 47 kDa cytosolic subunit of NADPH oxidase, a multicomponent enzyme that produces superoxide. Mice with genetic disruption for *NCF1* are more susceptible to *M. marinum* (Chao et al., 2017).

Abbreviations: aka, also known as; *MTB*, *Mycobacterium tuberculosis*; NTM, nontuberculous mycobacteria; PBMC, peripheral blood mononuclear cells.

**FIGURE 4:**
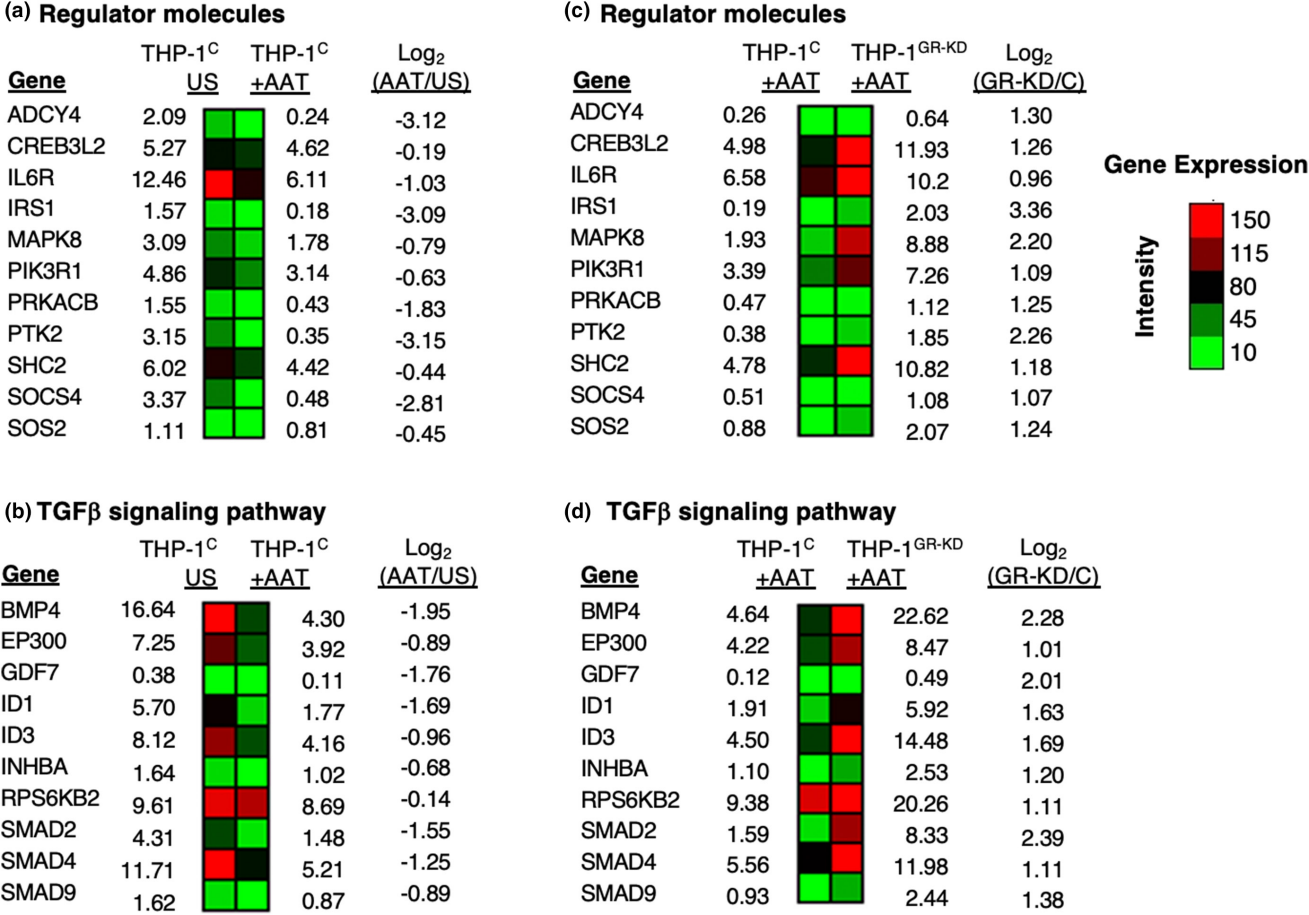
Heatmap of selected AAT‐inhibited, GR‐dependent genes. AAT‐inhibited gene expression with genes sorted by (a) regulator molecules and (b) TGFβ signaling pathway categories. (c, d) AAT‐inhibited, GR‐dependent gene expression sorted by the same categories as A and B. The numbers indicate Reads per Kilobase Million (RPKM). (a–d) represent duplicate of two biological samples. AAT, alpha‐1‐antitrypsin; C, control THP‐1 cells; GR‐KD, THP‐1 cells knocked down for glucocorticoid receptor; THP‐1^C^, control THP‐1 cells; THP‐1^GR‐KD^, THP‐1 cells knocked down for the glucocorticoid receptor; US, unstimulated.

We also categorized AAT‐downregulated, GR‐dependent genes into two pathways: (i) regulator molecules (Figure 4a,c) and (ii) transforming growth factor‐beta (TGFβ) signaling (Figure 4b,d). A brief description of some of the gene products that are AAT‐downregulated and GR‐dependent (shown in Figure 4a–d) and relevant for mycobacterial infections are shown in Table 2, whereas discussion of the functions of additional selected gene products are shown in Tables S8 and S9.

**TABLE 2:** AAT‐inhibited, GR‐dependent genes relevant for mycobacterial infections.

Gene	Protein	Brief synopsis of function
*GDF‐7*	Growth differentiation factor‐7	Encodes a secreted ligand that binds to various TGFβ receptors leading to recruitment and activation of SMAD family transcription factors that regulate gene expression (Hinck et al., 2016; Wan et al., 2021). While there is no direct link between GDF‐7 and mycobacterial infections, it is plausible that AAT inhibition of GDF‐7 expression is host‐protective since TGFβ is considered to predispose to mycobacterial infections due to its role in the regulation, deactivation, and suppression of cells (macrophages, dendritic cells, neutrophils, and cytotoxic T cells) that are important against infections (Batlle & Massagué, 2019; Ovrutsky et al., 2013; Warsinske et al., 2017; Wu et al., 2012).
*IL‐6R*	Cytokine 6 receptor (CD126)	IL‐6 is host‐protective against *MTB* in macrophages (Martinez et al., 2013) and in mice (Ladel et al., 1997). In mice, IL‐6 is important for both early induction of IFNγ and control of *MTB* infection but not essential for long‐term protective immunity (Saunders et al., 2000). IL‐6R also plays a role in the T_H_17 response (Zhang et al., 2019), which is host‐protective in early mycobacterial infection. Conversely, *MTB*‐induced IL‐6 in macrophages inhibited their responses to IFNγ (independent of any effects on STAT1 activation) (Nagabhushanam et al., 2003).
*SMAD4*	Suppressor of mothers against decapentaplegic homolog 4 transcription factor	SMAD4 is a downstream signaling molecule of TGFβ. Increased expression of TGFβ (and IL‐10) in the bronchoalveolar lavage cells of TB patients (compared to other lung diseases) may suppress effective anti‐TB immunity (Bonecini‐Almeida et al., 2004; David & Massagué, 2018). SMAD4 is crucial for differentiation of T_H_17 cells through IL‐21 (56).
*SOCS4*	*Suppressor of cytokine signaling 4*	SOCS are a group of proteins known to negatively regulate cytokine signaling. SOCS have also been found to inhibit effector T cell responses during *MTB* infection as well as contribute to resistance to IFNγ‐mediated mycobactericidal activity in human macrophages (Srivastava et al., 2011; Vázquez et al., 2006). However, SOCS4 in particular has been found to play a host‐protective role in trafficking of CD8 T cells and activation of T cell receptors during influenza infection (Kedzierski et al., 2014).

Abbreviations: IFNγ, interferon gamma; *MTB*, *Mycobacterium tuberculosis*; *SMAD4*, suppressor of mothers against decapentaplegic homologc‐4; *SOCS4*, *suppressor of cytokine signaling 4*; TGFβ, transforming growth factor‐beta.

### 3.3 Quantitation of selective AAT‐upregulated, GR‐dependent gene products

Since we have an interest in AAT as a host defense molecule against pathogens, we quantified both the mRNA of the following AAT‐upregulated, GR‐dependent genes by RT‐qPCR (*CSF2*, *IL‐1B*, *IL‐23A*, *IL‐27B*, *CCL1*, *CCL2*, *CCL3*, *CCL20*, *IFI6*, and *NCF1*) and protein by ELISA (GM‐CSF, IL‐1β, and IL‐27).

**FIGURE 5:**
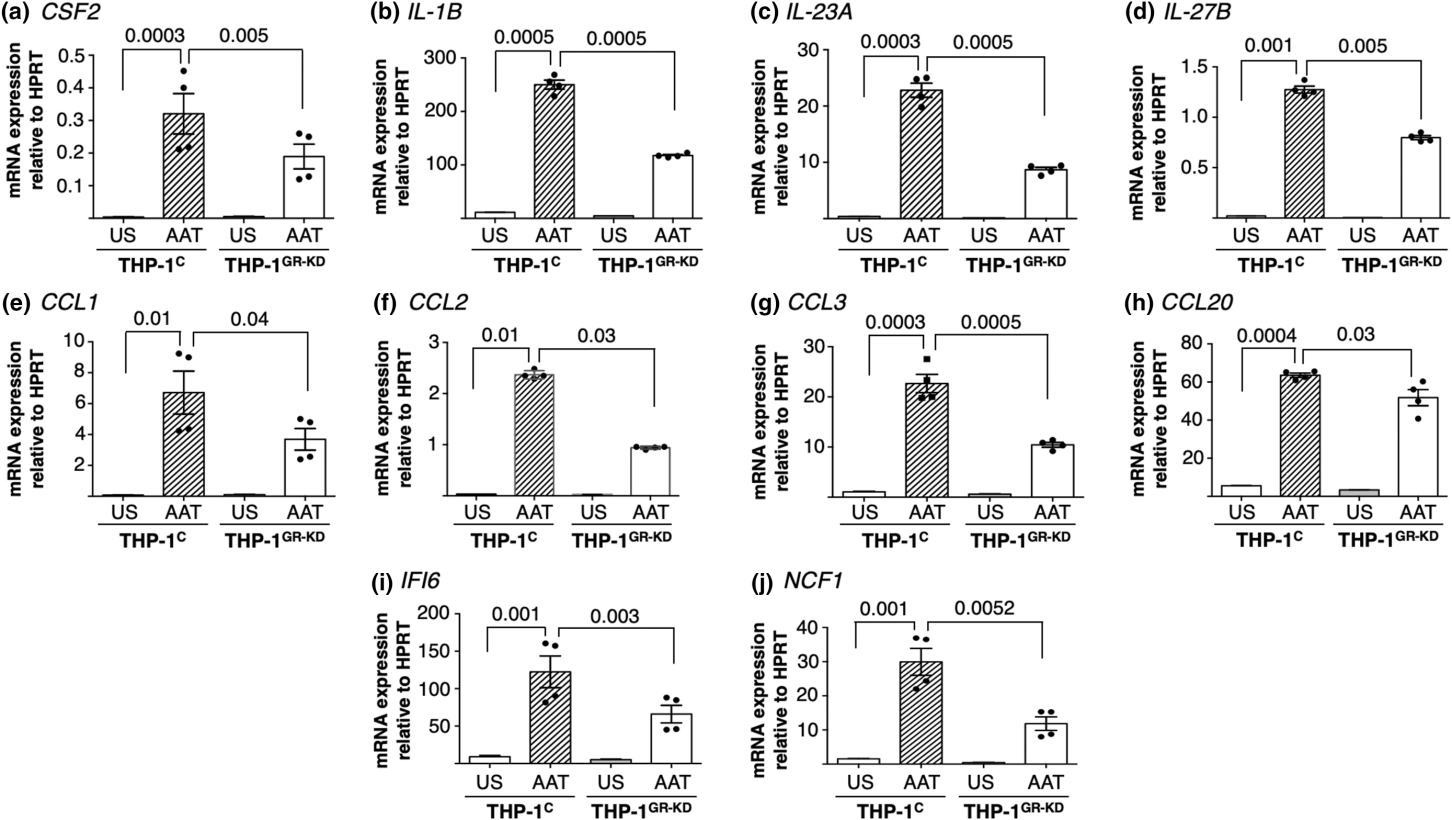
Quantitation of mRNA of selective AAT‐induced, GR‐dependent growth factors, cytokines, and chemokines. AAT‐induced, GR‐dependent gene expression were quantified by RT‐qPCR for: (a) *CSF2*, (b) *IL‐1B*, (c) *IL‐23A*, (d) *IL‐27B*, (e) *CCL1*, (f) *CCL2*, (g) *CCL3*, (h) *CCL20*, (i) *IFI6*, and (j) *NCF1*. *HPRT* was used as an endogenous (housekeeping) control in the comparative ΔΔ*C*_t_ method. Data shown are mean ± SEM of two independent experiments, each in duplicate samples. *p*‐Values are shown between indicated conditions. AAT, alpha‐1‐antitrypsin; *CSF2*, colony‐stimulating factor 2; *CCL*, C‐C motif chemokine ligand; *HPRT*, hypoxanthine phosphoribosyltransferase 1; *IFI6*, interferon alpha inducible protein 6; *IL‐1B*, interleukin‐1‐beta; *IL‐23A*, interleukin‐23 alpha subunit; *IL‐27B*, interleukin‐27 beta subunit; *NCF1*, neutrophil cytosolic factor 1; THP‐1^C^, control THP‐1 cells; THP‐1^GR‐KD^, THP‐1 cells knocked down for the glucocorticoid receptor; US, unstimulated.

The RT‐qPCR data for 10 of the AAT‐upregulated genes show that AAT strongly increased their expression by ≥9‐fold, consistent with the bulk RNA‐seq data (Figure 5a–j, the first and second bars of each graph). Compared to AAT‐stimulated THP‐1^control^ cells, THP‐1^GR‐KD^ cells stimulated with AAT showed a consistent reduction in their quantitative mRNA expression of the same 10 genes by 1.3‐3‐fold (Figure 5a–j, the second and fourth bars of each graph).

**FIGURE 6:**
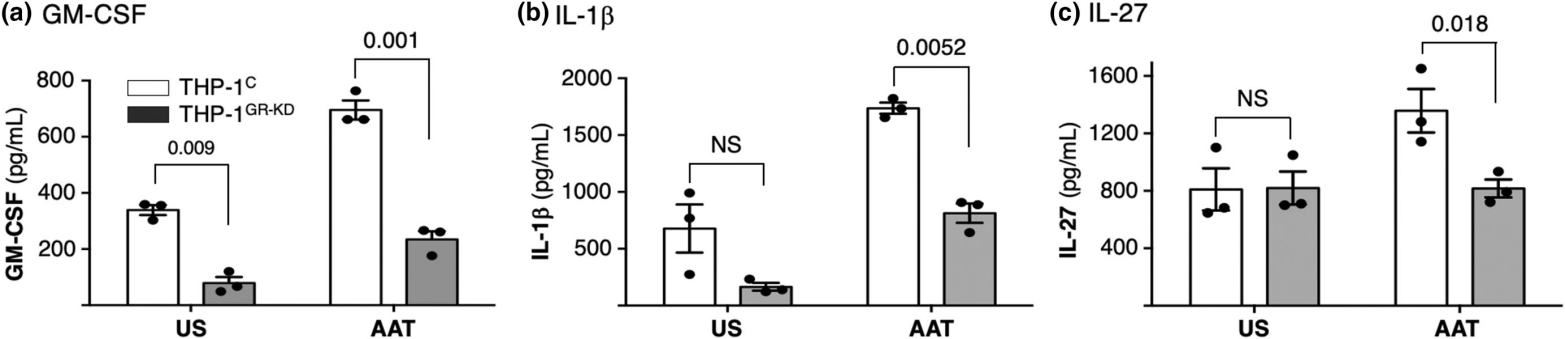
Quantitation of AAT‐induced, GR‐dependent expression of GM‐CSF, IL‐1β, and IL‐27 proteins. Differentiated THP‐1^control^ and THP‐1^GR‐KD^ macrophages were treated with or without AAT for 3 days, and the supernatants were collected and measured for (a) GM‐CSF, (b) IL‐1β, and (c) IL‐27 protein levels by ELISA. Data shown are mean ± SEM of three independent experiments with each performed in duplicate wells. *p*‐Values are shown between indicated conditions. GM‐CSF, granulocyte‐monocyte colony‐stimulating factor; IL‐1β, interleukin‐1‐beta; IL‐27, interleukin‐27; NS, not significant; THP‐1^C^, control THP‐1 cells; THP‐1^GR‐KD^, THP‐1 cells knocked down for the glucocorticoid receptor; US, unstimulated.

The ELISA data for GM‐CSF, IL‐1β, and IL‐27 qualitatively matched the bulk RNAseq, and RT‐qPCR data in that AAT induced the expression of GM‐CSF and IL‐1β by >2‐fold in the THP‐1^control^ cells and of IL‐27 by 1.6‐fold (Figure 6a–c, compare the open bars) and that these inductions were significantly less in the THP‐1^GR‐KD^ cells (Figure 6a–c, compare third and fourth bars). Basally (unstimulated), the levels of GM‐CSF and IL‐1β were less in the THP‐1^GR‐KD^ cells compared with the THP‐1^control^ cells (Figure 6a,b, compare first two bars of each graph); this was not seen with IL‐27 (Figure 6c, compare first two bars).

### 3.4 Quantitation of selective AAT‐downregulated, GR‐dependent transcripts

**FIGURE 7:**
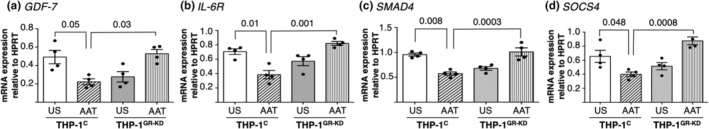
Quantitation of mRNA of selective AAT‐inhibited, GR‐dependent genes. AAT‐inhibited, GR‐dependent gene expression were quantified by RT‐qPCR of (a) *GDF‐7*, (b) IL‐6R, (c) *SMAD4*, and (d) *SOCS4*. *HPRT* was used as an endogenous (housekeeping) control in the comparative ${\Delta \Delta C}_{t}$ method. Data shown are mean ± SEM of two independent experiments, each in duplicate samples. *p*‐Values are shown between indicated conditions. AAT, alpha‐1‐antitrypsin; *GDF‐7*, growth differentiation factor‐7; *HPRT*, hypoxanthine phosphoribosyltransferase 1; *IL‐6R*, Cytokine 6 Receptor (CD126); *SMAD4*, suppressor of mothers against decapentaplegic homolog 4; *SOCS4*, suppressor of cytokine signaling 4; THP‐1^C^, control THP‐1 cells; THP‐1^GR‐KD^, THP‐1 cells knocked down for the glucocorticoid receptor; US, unstimulated.

For AAT‐downregulated, GR‐dependent genes, we quantified the mRNA of the following genes: *GDF‐7* (growth differentiation factor‐7), *IL‐6R* (interleukin‐6 receptor), *SMAD4* (suppressor of mothers against decapentaplegic homolog 4), and *SOCS4* (suppressor of cytokine signaling 4). The RT‐qPCR data for all four transcripts showed that AAT significantly inhibited their expression (Figure 7a–d, compare first two bars), consistent with bulk RNA‐seq data (Figure 4a). However, compared to AAT downregulation of GDF‐7, IL‐6R, SMAD4, and SOCS4 mRNA in THP‐1^control^ cells, there was significant abrogation of their downregulation by AAT in THP‐1^GR‐KD^ cells (Figure 7a–d, compare second and fourth bars).

## 4 DISCUSSION

In addition to its canonical serpin function of inhibiting elastase and other serine proteases, AAT has a panoply of other noncanonical functions including: (i) augmenting host immunity against various pathogens including influenza (Harbig et al., 2020), HIV (Bryan et al., 2010; Shapiro et al., 2001; Whitney et al., 2011; Zhou et al., 2011, 2016), SARS‐CoV‐2 (Azouz et al., 2021; Bai et al., 2021; Bai, Buckle, et al., 2022; McElvaney et al., 2020; Ritzmann et al., 2021; Shapira et al., 2020; Wettstein et al., 2021), *Pseudomonas aeruginosa* (Pott et al., 2013), *M. intracellulare* (Bai, Bai, et al., 2022), and *Streptococcus pneumoniae* (Ostermann et al., 2021); (ii) mitigating against excessive and injurious inflammation (Bai et al., 2019; Bai, Bai, et al., 2022; Jonigk et al., 2013; Lewis, 2012; Wanner et al., 2012); (iii) protecting against epithelial and endothelial cell injury and inhibiting apoptosis (Greene et al., 2010; Petrache, Fijalkowska, Medler, et al., 2006); and (iv) improving allograft survival in cellular and murine models of allograft rejection (Conrad et al., 2017; Iskender et al., 2016; Kleinerova et al., 2019; Lewis et al., 2005, 2008; Lin et al., 2018; Marcondes et al., 2011; Pileggi et al., 2008; Shahaf et al., 2011; Tawara et al., 2012). While the mechanisms for some of these AAT functions are related to its serpin activity, others are related to its nonserpin activities, possibly functioning as a carrier protein for other molecules or a more direct active role (O'Brien et al., 2022). Indeed, in addition to binding GR, AAT may also contain a glucocorticoid‐binding domain (Huber & Carrell, 1989; O'Brien et al., 2022).

Through regulation of gene expression, AAT functions to mediate host defense and anti‐inflammatory properties. However, the mechanism by which AAT affects gene regulation remains poorly understood. Since the GR—a nuclear receptor regulation family of transcriptional regulators—is capable of binding an array of chaperone molecules and various transcriptional coactivators and corepressors as well as having overlapping anti‐inflammatory functions as AAT, we explored the possibility that AAT may regulate genes through GR. GR normally resides in the cytoplasm, but upon binding to endogenous or exogenous glucocorticoid, the glucocorticoid–GR complex translocates to the nucleus to control the expression of a wide variety of genes that regulate metabolism, inflammation, and host immunity. We recently reported that the AAT–GR complex affects cell signaling and gene expression, including the inhibition of both lipopolysaccharide‐induced NFκB activation and IL‐8 production (Bai, Bai, et al., 2022). Schuster and coworkers (Schuster et al., 2020) showed in various cell models that while both AAT and the glucocorticoid dexamethasone were anti‐inflammatory, AAT enhanced lipopolysaccharide‐induced IL‐1 receptor antagonist and re‐epithelization, whereas dexamethasone suppressed these specific anti‐inflammatory and reparative processes of AAT. Furthermore, while AAT did not antagonize anti‐inflammatory activities of dexamethasone, dexamethasone at medium to high levels antagonized the beneficial effects of AAT (Schuster et al., 2020). The specific mechanisms by which the glucocorticoid–GR and AAT–GR complexes interact with each other during transcription remains to be determined but are likely to be variable, depending not only on the specific gene involved but also on the intracellular and intranuclear milieu, dictated by the inflammatory/activation state of the cell(s).

The processes by which AAT mediates host defense are varied; example, we found that AAT reduced intracellular burden of *M. intracellulare* in macrophages through induction of autophagy (Bai et al., 2019). We and others have also found that AAT inhibits TMPRSS2, the cell surface serine protease that is normally required to cleave the spike protein of SARS‐CoV‐2, a necessary processing step prior to viral entry (Hoffmann et al., 2020). Herein, we found that AAT, which enhances host innate immunity against mycobacteria, induced several cytokines (IL‐1β, IL‐23) and a growth factor (GM‐CSF) that also play a host‐protective role against various microbial pathogens including mycobacteria (Abdul‐Rahman et al., 2004; Bai et al., 2019; Chan et al., 2007; De Groote et al., 2014; Witty et al., 1994). While we previously found that AAT inhibited lipopolysaccharide‐induced IL‐8 production in a GR‐dependent fashion, we show in the current study that AAT induced the expression of *IL‐23A* and *IL‐27B* genes, suggesting, by mechanisms that remain to be determined, that AAT alone may induce host‐protective cytokines but is also able to dampen the effects of an inflammatory stimulus like lipopolysaccharide. In this macrophage model, a significant part of AAT induction of GM‐CSF depended on GR, but there is likely a component that is independent of GR. We discuss below the significance of GM‐CSF in the context of mycobacterial infections given its induction by AAT.

GM‐CSF is produced by several cell types including type 2 alveolar epithelial cells, myeloid cells (monocytes, macrophages, dendritic cells, neutrophils, eosinophils), lymphocytes (T cells, B cells, NK cells), and fibroblasts (Louis et al., 2020; Mishra et al., 2020; Rothchild et al., 2017; Ushach & Zlotnik, 2016). GM‐CSF also skews monocytes toward an M1‐like macrophage phenotype, which can be further polarized to M1 macrophages with IFNγ and lipopolysaccharide; in contrast, monocyte colony‐stimulating factor (M‐CSF) polarizes monocytes toward an M2‐like phenotype (Mily et al., 2020). Hence, reduced GM‐CSF activity impairs differentiation of M1 macrophages and IFNγ‐producing T_H_1 cells, resulting in reduced ability to control mycobacterial infections (Mishra et al., 2020).

GM‐CSF plays a protective role against various infectious agents including *S. pneumoniae*, *P. aeruginosa*, mycobacteria, fungi, influenza, and SARS‐CoV2 (Ballinger et al., 2006; Chen et al., 2023). Interestingly, these are essentially the same organisms that AAT antagonizes. Blocking GM‐CSF increased *Mycobacterium tuberculosis* (*MTB*) burden in primary human macrophages, whereas adding GM‐CSF attenuated the *MTB* burden (Bryson et al., 2019). Mishra and colleagues (Mishra et al., 2020) demonstrated that compared to murine macrophages, human macrophages produced more GM‐CSF and greater expression of genes involved in GM‐CSF signaling pathway and that these increases correlated with better control of *MTB* infection in the macrophages. In a follow‐up study, the same investigators found that in human macrophages, greater levels of secreted GM‐CSF correlated with macrophage survival, a stronger in vitro granuloma‐like response, increased production of IL‐1β, IL‐12, and IL‐10, decreased levels of TNF and IL‐6, and reduced intracellular burden of *MTB*; conversely, depletion of GM‐CSF increased macrophage cell death and decreased autophagy (Mishra et al., 2022). Other mechanisms by which GM‐CSF may antagonize *MTB* in infected macrophages include increased phagosome‐lysosome fusion (Pasula et al., 2015) and nitric oxide production (Benmerzoug et al., 2018). In coculture of invariant natural killer T (iNKT) cells and macrophages, production of GM‐CSF by the iNKT cells enhanced control of *MTB* infection in the macrophages (Rothchild et al., 2014). Gail and co‐workers (Gail et al., 2023) showed in primary human macrophages and T cells that *MTB*‐infected macrophages that were differentiated with GM‐CSF (more M1‐like) were better able to activate T cells than *MTB*‐infected macrophages that were differentiated with M‐CSF, which were more M2‐like. Such dichotomy is likely an oversimplification as there are many genes that are induced or inhibited in macrophages in a similar fashion by GM‐CSF or M‐CSF (Martinez & Gordon, 2014). Further supporting the importance of GM‐CSF in host defense against mycobacteria is the finding that a chronic *Mycobacterium abscessus* lung infection has been established in the GM‐CSF knockout mice (De Groote et al., 2014).

It is likely that both GM‐CSF and M‐CSF are important in host defense against mycobacteria as these two CSFs activate different populations of immune cells, with GM‐CSF differentiating dendritic cells and priming of macrophages and NK cells, whereas M‐CSF is important for mobilization of myeloid cells into the blood and subsequently into tissue, as well as maintenance of specific macrophage populations to maintain tissue integrity (Hamilton, 2008). Furthermore, orchestration of the temporal kinetics and plasticity of the different macrophage phenotypes during the initial encounter with the infection (with GM‐CSF‐derived M1‐like macrophages) and subsequent resolution of the inflammation (with M‐CSF‐derived M2‐like macrophages) is likely crucial for effective control of the overall infection. In this regard, it is interesting that GM‐CSF induces M‐CSF production in monocytes and macrophages, suggesting that sequential expression of these two growth factors may be optimal for host defense against mycobacterial infections (Benmerzoug et al., 2018; Martinez & Gordon, 2014).

The relative amounts of M‐CSF and GM‐CSF have also been studied in the context of murine model of *MTB* infection. Higgins et al. (2008) found a progressive decrease in M‐CSF and an increase in GM‐CSF in the course of *MTB* infection of mice. Repleting M‐CSF during *MTB* infection decreased the number of foamy macrophages, increased class II MHC molecules on the alveolar macrophages, increased expression of the pattern‐recognition receptor DEC‐205, and increased their T cell stimulating capacity. This activating effect of M‐CSF on T cells in mice (Higgins et al., 2008) is in contradistinction to that seen in primary human cells (Gail et al., 2023). However, others implicate GM‐CSF as being more protective than M‐CSF in mice against mycobacterial infections. In TNF‐deficient mice, anti‐GM‐CSF antibody (that neutralizes GM‐CSF) impaired control of *MTB* infection with exacerbated lung inflammation and necrotic granulomas (Benmerzoug et al., 2018). These investigators further showed that in vitro, anti‐GM‐CSF antibody skewed *Mycobacterium bovis* BCG‐infected macrophages toward the “immunosuppressive” M2 phenotype with decreased nitric oxide production and increased intracellular burden of BCG (Benmerzoug et al., 2018). *MTB* infection of mice engineered for GM‐CSF deficiency had impaired granuloma formation and were more susceptible (Szeliga et al., 2008).

**FIGURE 8:**
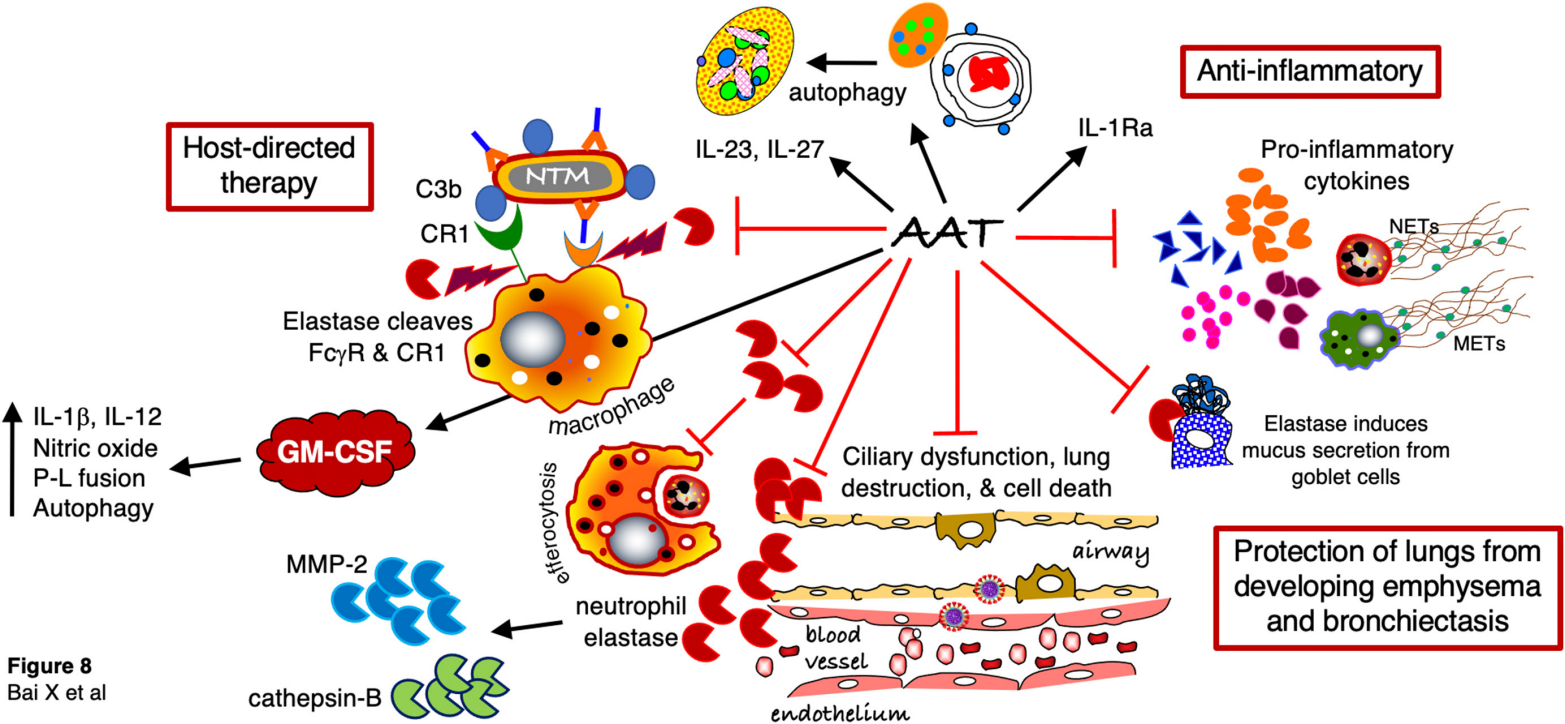
Diagram of the various host‐protective function of AAT against mycobacteria. Starting from the left upper diagram and going clockwise, AAT: (i) inhibits elastase cleavage of the Fcγ receptor and the complement receptor 1 (CR1); (ii) induces host‐protective cytokines IL‐23 and IL‐27 as well as autophagy in macrophages, an intracellular effector mechanism against mycobacteria; (iii) attenuates excessive inflammation and mucus hypersecretion; (iv) protects the lungs from elastase‐mediated ciliary dysfunction, lung destruction (emphysema), and cell death (bronchiectasis), substrates for mycobacterial infections, by inhibiting neutrophil elastase and downstream proteases such as MMP‐2 and cathepsin‐B; (v) mitigates elastase‐mediated inhibition of efferocytosis (impairing clearance of dead neutrophils, inciting injurious inflammation through release highly viscous DNA that also augments the volume of inspissated mucus), and (vi) induces GM‐CSF, an important differentiating factor for host‐protective macrophages against mycobacteria by several mechanisms including induction of IL‐1β, IL‐12, nitric oxide, P‐L fusion, and autophagy. C3b, complement component 3b; CR1, complement receptor‐1; FcγR, cell surface receptor that binds constant region of immunoglobulin G; GM‐CSF, granulocyte monocyte‐colony‐stimulating factor; IL‐Ra, IL‐1 receptor antagonist; METs, macrophage extracellular traps; MMP‐2, matrix metalloproteinase‐2; NETs, neutrophil extracellular traps; NTM, nontuberculous mycobacteria; P‐L, phagosome‐lysosome. 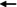 = induces; 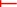 = inhibits; 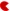 = neutrophil elastase.

The essential role of GM‐CSF in housekeeping macrophage function is evinced by the accumulation of lipoproteinacious material (surfactant) with defects in GM‐CSF function, which is characteristic of a clinical disorder known as pulmonary alveolar proteinosis (PAP). While there are congenital and secondary forms of PAP, the most common (or at least the most recognizable) is the primary form of PAP, which results from the presence of autoimmune antibodies directed against GM‐CSF (Trapnell et al., 2003). This neutralization of GM‐CSF results in a defect in macrophages' ability to dispose of ingested material, apparent in their inability to degrade phagocytosed surfactant. Hence, for the same likely reason, PAP patients are vulnerable to infections such as *Nocardia*, fungi, and NTM such as *Mycobacterium kansasii* and *M. avium* complex because their macrophages are compromised (Abdul‐Rahman et al., 2004; Bakhos et al., 1996; Bedrossian et al., 1980; Carnovale et al., 1977; Goldschmidt et al., 2003; Prakash et al., 1987; Ramirez, 1967; Witty et al., 1994). Interestingly, in non‐PAP patients with NTM lung disease, it was found that the mean anti‐GM‐CSF antibody, anti‐IFNγ antibody, and total IgG were higher than controls, suggesting the possibility that even in those without autoimmune PAP, higher levels of anti‐GM‐CSF antibody (and anti‐IFNγ antibody) may play a role in increasing the vulnerability to NTM lung disease (Kim et al., 2014). Inhaled GM‐CSF alone or with antibiotics showed efficacy against *M. abscessus* lung infection in cystic fibrosis patients (Scott et al., 2018; Thomson et al., 2024). Our finding that AAT induces expression of GM‐CSF indicate that the host defense mechanism of AAT against mycobacteria may occur by several mechanisms including prevention of elastase from cleaving Fcγ receptor‐1 and complement receptor‐1 from cell surface, induction of autophagy, prevention of excessive inflammation and mucus hypersecretion, prevention of ciliary dysfunction and structural lung injury such as emphysema and bronchiectasis that predispose to infection, prevention of elastase inhibition of efferocytosis, and induction of GM‐CSF (Bai et al., 2019; Bai, Bai, et al., 2022; Bergin et al., 2010; Lewis, 2012; Petrache, Fijalkowska, Medler, et al., 2006; Petrache, Fijalkowska, Zhen, et al., 2006; Serban & Petrache, 2016; Tosi et al., 1990; Tosi & Berger, 1988) (Figure 8).

A limitation to this study is that differentiated THP‐1 cells were used and thus, the findings are unlikely to be applicable across all human subjects based on differences in genotypes. However, this same limitation is also a strength because by employing THP‐1 cells, we have been able to create a stable cell population of THP‐1^control^ and THP‐1^GR‐KD^ cells; such a stable knockdown has an inherently greater transfection efficiency of the lentivirus shRNA targeting GR than transient knockdown of GR in primary macrophages. Furthermore, since we wish to compare control cells with cells knocked down for GR, using the THP‐1 cell line keeps other variables to a minimum; that is, primary macrophages, even obtained from the same individual, are more likely to have chronotropic fluctuations of the immune cell phenotype. In addition, we and others have been shown that the THP‐1 cell line mimics qualitatively the responses of primary human macrophages to different stimuli, particularly with mycobacterial infections (Bai et al., 2011, 2013; Riendeau & Kornfeld, 2003; Shang et al., 2011; Stokes & Doxsee, 1999; Theus et al., 2004).

While we have shown that AAT induction of both GM‐CSF mRNA and protein is significantly dependent on GR, it is interesting that glucocorticoids, via GR, have been described to inhibit GM‐CSF expression at the pre‐transcriptional, transcriptional, and posttranscriptional/translational levels (Adkins et al., 1998; Bergmann et al., 2004; Newton et al., 2010; Smith et al., 2001; Tobler et al., 1992). More specifically, glucocorticoid GR have been shown to: (i) increase GM‐CSF mRNA degradation (Tobler et al., 1992); (ii) induce a phosphatase (mitogen‐activated protein kinase phosphatase‐1) that inhibits activation (phosphorylation) of extracellular signal‐regulated kinase (ERK), which mediates IL‐1β‐induced GM‐CSF (Lehtola et al., 2022; Newton et al., 2010); (iii) compete with transcriptional activator (NFAT‐AP‐1) induction of *CSF‐2* (GM‐CSF) gene expression by phorbol dibutyrate plus ionomycin in T cells (Smith et al., 2001); and (iv) reduce release of GM‐CSF protein (Bergmann et al., 2004). By another mechanism that is more indirect, IL‐1α activation of p38^*mapk*^ stabilizes GM‐CSF mRNA and increases GM‐CSF protein production, while dexamethasone inhibits p38^*mapk*^ activation, leading to reduced amount of GM‐CSF protein (Tran et al., 2005). All these and our current findings indicate a dichotomous relationship between glucocorticoid–GR and AAT–GR on GM‐CSF expression, a paradigm that paves the way for future studies since glucocorticoids are known to predispose to mycobacterial infections whereas AAT is protective. On the other hand, whereas AAT and dexamethasone have opposing effects on GM‐CSF expression, we found that AAT and dexamethasone cooperated to inhibit lipopolysaccharide‐induced IL‐8 expression (Bai, Bai, et al., 2022), indicating that AAT and glucocorticoids may either cooperate or antagonize each other depending on the gene being regulated. Another unanswered question is the seeming paradox that AAT marshals host defense activities against microbial pathogens despite the fact that elastase, which is irreversibly inhibited by AAT, is also an antimicrobial molecule (Stapels et al., 2015). One plausible explanation is that in phagocytes, *cytoplasmic* AAT bound to GR—while maintaining its host defense function through gene regulation—may be less capable of neutralizing elastase, which is then available to antagonize intracellular pathogens. Yet, AAT secreted extracellularly, not bound to intracellular GR, maintains its ability to neutralize *extracellular* elastase, limiting tissue damage.

## CONFLICT OF INTEREST STATEMENT

All authors declare that they have no conflict of interest with the contents of this article.

## ETHICS STATEMENT

The authors are accountable for all aspects of the work in ensuring that questions related to the accuracy or integrity of any part of the work are appropriately investigated.

## Supporting information


Tables S1–S9.

## Data Availability

Raw data are available for review.
