# Supplementary material for: Analysis of alpha‐1‐antitrypsin (AAT)‐regulated, glucocorticoid receptor‐dependent genes in macrophages reveals a novel host defense function of AAT
Source: Physiol Rep. 2024 Jul 17;12(14):e16124. doi: 10.14814/phy2.16124 (PMC11252833; doi:10.14814/phy2.16124)
Supplement: Supplementary file 1 — Tables S1–S9. [file PHY2-12-e16124-s001.zip › phy216124-sup-0001-DataS1.docx]

**Supplemental Tables**

**Supplemental Table 1. Primer sequences for the RT-qPCR**

| **Gene name** | **Forward sequence (5’ to 3’)** | **Reverse sequence (5’ to 3’)** |
| --- | --- | --- |
| ***CSF2*** | GGAGCATGTGAATGCCATCCAG | CTGGAGGTCAAACATTTCTGAGAT |
| ***IL-23A*** | GAGCCTTCTCTGCTCCCTGATA | GACTGAGGCTTGGAATCTGCTG |
| ***IL-1B*** | CCACAGACCTTCCAGGAGAATG | GTGCAGTTCAGTGATCGTACAGG |
| ***IL-27B*** | CTGGATCCGTTACAAGCGTCAG | CACTTGGACGTAGTACCTGGCT |
| ***CCL1*** | ACCAGCTCCATCTGCTCCAATG | TGTGCCTCTGAACCCATCCAAC |
| ***CCL2*** | AGAATCACCAGCAGCAAGTGTCC | TCCTGAACCCACTTCTGCTTGG |
| ***CCL3*** | ACTTTGAGACGAGCAGCCAGTG | TTTCTGGACCCACTCCTCACTG |
| ***CCL20*** | AAGTTGTCTGTGTGCGCAAATCC | CCATTCCAGAAAAGCCACAGTTTT |
| ***IFI6*** | TGATGAGCTGGTCTGCGATCCT | GTAGCCCATCAGGGCACCAATA |
| ***NCF1*** | AATGGCAGGACCTGTCGGAGAA | CCTGTTCTCTGGATTGATCGCC |
| ***IL-6R*** | GACTGTGCACTTGCTGGTGGAT | ACTTCCTCACCAAGAGCACAGC |
| ***GDF-7*** | TGATGTCGCTTTACCGGAGC | TGGACACGTCGAACAGGAAG |
| ***SOCS4*** | GGGTAAGCACAGACTTGTCTCAG | TCACAGAGCCAGTCATAGGACC |
| ***SMAD4*** | CTACCAGCACTGCCAACTTTCC | CCTGATGCTATCTGCAACAGTCC |
| ***HPRT1*** | CTCATGGACTAATTATGGACAGGAC | GCAGGTCAGCAAAGAATTTATAGCC |

**Supplemental Table 4. AAT-induced, GR-dependent cytokine and chemokine genes**

| **Gene** | **Protein** | **Selected key function*** |
| --- | --- | --- |
| ***CXCL13*** | Chemokine C-X-C motif ligand 13 (CXCL13); binds to receptor CXCR5. | Produced mainly by follicular dendritic cells. CXCL13 is a chemoattractant for B and T cells, important for granuloma formation, and control of *MTB* infection (1). |
| ***GDF15*** | Growth Differentiation Factor 15 | A secreted ligand of the TGFβ superfamily of protein. GDF15 has been shown to influence the activity of macrophages, namely by suppressing their ability to produce TNF (2) as well as by promoting their ability to produce antimicrobial peptides, such as LL-37 (3). GDF15 has also been found to play a role in the T cell response to infection, namely by inhibiting the proinflammatory Type 1 response and promoting the anti-inflammatory Type 2 response (4). |

*Preferential focus on host-defense function against mycobacterial (microbial) pathogens

**Supplemental Table 5. AAT-induced, GR-dependent kinase and regulator molecule genes**

| **Gene** | **Protein** | **Selected key function*** |
| --- | --- | --- |
| ***CD40*** | CD40 | A member of the TNF receptor superfamily, CD40 activates downstream cytokines and chemokines (5). CD40 ligand – alone or in combination with IFNγ – activates the vitamin D-dependent induction of antimicrobial peptides cathelicidin and DEFB4 as well as induction of autophagy (6). CD40 engagement on dendritic cells induces the Notch ligand DLL4, necessary for differentiation of T_H_17 cells, which provides host-protection during early phase of *MTB* infection (7). |
| ***MDM2*** | E3 ubiquitin ligase | A negative regulator of the p53 tumor suppressor (8, 9). Since p53 increases apoptosis of *MTB*-infected macrophages and reduces *MTB* burden (10), AAT-GR induction of MDM2 may increase vulnerability of the host against *MTB*. |
| ***IL1RN*** | IL-1 receptor antagonist (IL-1Ra) | An anti-inflammatory protein that antagonizes the receptor to IL-1 (11). While IL-1 confers protection against *MTB* (12), IL-1Ra may be necessary to dampen inflammation once the infection is under controlled. |
| ***FOSL1*** | Fos | A subunit of the AP-1 transcription factor, involved in widespread transcriptional activation (13). FOSL1 and FOSL2 co-repress differentiation of T_H_17 lineage (14). |
| ***SPHK1*** | Sphingosine Kinase 1 (SphK-1) | Regulates tumor cell proliferation and apoptosis (15). SphK-1 increased macrophage ability to control *Mycobacterium smegmatis* infection through induction of nitric oxide (16). |
| ***PLA2G6*** | Phospholipase A2 (PLA2) Group VI | Intracellular enzymes that catalyze the hydrolysis of lipid mediators and involved in biological processes including cell differentiation, signal transduction, and apoptosis (17). Whereas cytosolic PLA2 (group IV) mediates apoptosis of *MTB*-infected human macrophages and thus host-protective, cytosolic PLA2 (group VI) does not mediate such apoptosis (18). |
| ***ICAM1*** | Intercellular Adhesion Molecule 1 | Regulator of many inflammatory processes, including leukocyte trafficking, efferocytosis, and T-cell activation (19). ICAM1 is required for both TGFβ-induced FoxP3 expression and T regulatory cell expression, which is important to prevent excessive inflammatory pathology in *MTB*-infected mice (20). |
| ***PLAUR*** | Urokinase plasminogen activator receptor | Promotes cell survival, migration and resistance to targeted cancer therapeutics (21). Promotes macrophage differentiation, an important response to mycobacterial infection (22). Also required for chemotaxis in mononuclear phagocytes, which are cells important during the mycobacterial response (23). |
| ***CDKN1A*** | Cyclin-dependent kinase inhibitor 1A (p21, Cip1) | Regulates cell growth and cellular response to DNA damage (24). May inhibit apoptosis (25). Facilitates in the formation and maturation of sarcoid granulomas (26). |
| ***RIPK2*** | Receptor-interacting serine/threonine-protein kinase 2 | In macrophages, *MTB* induces the Nod2/Rip2/Irf5 pathway that upregulates type 1 interferon expression (27). |

*Preferential focus on host-defense function against mycobacterial (microbial) pathogens

**Supplemental Table 6. AAT-induced, GR-dependent apoptosis and TNF signaling genes**

| **Gene** | **Protein** | **Selected key function*** |
| --- | --- | --- |
| ***PIM1*** | Serine/threonine-protein kinase pim-1 | Proto-oncogene with serine/threonine kinase activity involved in cell survival and cell proliferation (28). |
| ***CTSH*** | Cathepsin H | A lysosomal cysteine protease involved with maturation and stabilization of Toll-like receptor 3 and subsequent interferon-beta production (29). Interestingly, clofazimine or rifamycin inhibits cathepsin H activity with the potential to attenuate mycobacteria-induced tissue damage (30). |
| ***PPIF*** | Cyclophilin F; aka cyclophilin D | Cyclophilin F (a peptidylprolyl isomerase) is part of the permeability transition pore in the inner mitochondrial membrane, activation of which induces apoptotic and necrotic cell death (31). Cyclophilin D, as a checkpoint of T cell metabolism, limits expansion of T cells but mice with cyclophilin D-deficient T cells (paradoxically) had compromised disease tolerance with greater vulnerability to *MTB* (32). The glycopeptidolipids of *M. abscessus* inhibits macrophage apoptosis (with subsequent inhibition of intracellular growth / mycobacterial spread) by interacting (deactivating) cyclophilin D (33). |
| ***BCL3*** | B cell chronic lymphatic leukemia protein 3 | Regulates pro-survival and pro-inflammatory gene expression in cutaneous T-cell Lymphoma (34). Increased BCL-3 expression reduced apoptosis of *MTB*-infected human alveolar macrophages with presumed reduced control of *MTB* infection (35). |
| ***SGK1*** | Serum/glucocorticoid regulated kinase 1 (SGK1) | SGK1 helps mediate IL-23 receptor development of T_H_17 cells and inhibition of T regulatory cells (36). |
| **TMEM214** | Transmembrane protein 214 | Mediates endoplasmic reticulum stress-induced caspase 4 enzyme activation and apoptosis (37). ER stress-induced apoptosis of macrophages found in TB granulomas, which may be either protective (by killing intracellular MTB) but may provide a mode for dissemination (in advanced granulomas) (38). |
| ***MMP9*** | Matrix metalloproteinase-9 (MMP-9) | Elevated plasma levels of MMP-9 found in patients with pulmonary TB compared to matched uninfected controls (39). In human pleural mesothelial cells, *MTB* induced MMP-9 expression (40). Since MMP-9 helps degrade the extracellular matrix, it may help spread mycobacterial infection. |
| ***MAP1S*** | microtubule-associated protein 1S | Regulates the phagocytosis of bacteria and Toll-like receptor (TLR) signaling (41) and enhances autophagy (42). |
| ***IGFBP3*** | Insulin-like growth factor binding protein 3 (IGFBP-3) | IGFBP-3 negatively regulates NFκB signaling (43). Inhibition of NFκB in macrophages has been shown to induce autophagy in macrophages against *MTB* (44). |
| ***HMOX1*** | Heme Oxygenase-1 (HO-1) | HO-1 degrades free heme to carbon monoxide and biliverdin, which have both anti-inflammatory and ability to restrict pathogen growth (45, 46). |
| ***DIABLO*** | Diablo IAP-binding mitochondrial protein | Diablo promotes apoptosis by binding to IAPs (inhibitors of apoptosis) and preventing them from inhibiting caspases (47). |
| ***PIM3*** | Serine/threonine-protein kinase pim-3 | A proto-oncogene that inhibits apoptosis, sustains cell survival, and regulates the progression of the cell cycle (48). PIM3 appears to inhibit differentiation of T_H_17 cells (49). |
| ***DDIT4*** | RTP801 (aka REDD1 or Dig2) | RTP801 is known to inhibit mTOR (mammalian target of rapamycin) activity (50), which, in turn, should activate autophagy. Hypoxia activates HIF-1 (hypoxia-inducible factor-1) transcription factor, which induces expression of *DDIT4* gene, the product of which may enhance apoptosis (51). Since hypoxia is a common characteristic found in TB granulomas, activation of RTP801 may enhance both autophagy and apoptosis, two macrophage effector mechanisms against intracellular mycobacteria. |

*Preferential focus on host-defense function against mycobacterial (microbial) pathogens

**Supplemental Table 7. AAT-induced, GR-dependent antiviral and anti-mycobacterial genes**

| **Gene** | **Protein** | **Selected key function*** |
| --- | --- | --- |
| ***ZC3H12A*** | MCPIP-1 (monocyte chemoattractant protein 1-induced protein-1) | MCPIP-1 attenuates lipopolysaccharide-induced NFκB activation as well as TNF and inducible nitric oxide synthase promoter activities (52, 53). Plays an important role in degradation of IL-6, which has both host-protective and host-damaging effects with *MTB* infection (54, 55) |
| ***CEBPB-AS1*** | CCAAT/enhancer-binding protein beta antisense RNA | Inhibits expression of the transcription factor CEBPB (56). C/EBPβ signaling axis also favors M2 polarization, which may impair clearance of *MTB* during active infection but can help resolve the inflammation once *MTB* infection is controlled (57). |
| ***ISG15*** | A 17 kDa secreted ubiquitin-like protein | Induces IFNγ secretion and possesses direct anti-viral activities, in part, by modifying substrates in a process similar to ubiquitination (ISGylation) (58). ISG15 deficiency leads to increased type 1 interferons (IFNα/β) and decreased IFNγ; such individuals are more susceptible to mycobacterial infections (59, 60). |
| ***FCGR1A*** | High-affinity IgG Fc receptor 1 (aka CD64) | Plays an important role in the immune response through cell phagocytosis, clearance of immune complexes, and activation and regulation of certain immune cells (61). In children with active TB, FCGR1A transcript correlated with TB severity and decreased with TB treatment (62). Whole blood RNA analysis showed that the level of gene product of FCGR1A strongly correlated with active disease (63). |
| ***PLSCR1*** | Human phospholipid scramblase 1 | Strongly expressed in response to interferon treatment and viral infection, it may have been suggested to play an important role in IFN-dependent antiviral response (64). |
| ***IFITM3*** | Interferon-induced transmembrane protein 3 | A member of the IFITM protein family, IFITM3 protein blocks viral entry and has been found to restrict intracellular growth of *MTB* by enhancing endosomal acidification (65). |
| ***IFITM1*** | Interferon-induced transmembrane protein 1 | IFITM1 protein blocks viral entry and has been found to restrict intracellular growth of *MTB* (65). |
| ***IRF7*** | Interferon regulatory factor 7 | A transcription factor that is induced by various pathogens (especially viruses such as Epstein-Barr virus) and is critical to the production of type I interferons (66, 67). IRF-7, through induction of miRNA-31, protects against *MTB* infection in mice *via* reduction in both *MTB* burden and severity of lung pathology (68). |

*Preferential focus on host-defense function against mycobacterial (microbial) pathogens

**Supplemental Table 8. AAT-inhibited, GR-dependent regulator molecule genes**

| **Gene** | **Protein** | **Selected key function*** |
| --- | --- | --- |
| ***ADCY4*** | Adenylate Cyclase 4 | An enzyme that catalyzes the formation of cAMP and has been found to be involved in pathways involved in tumorigenesis (69). cAMP inhibits LPS-induced inflammasome activation by inhibiting caspase-11-mediated proteolytic maturation of IL-1β (70). Thus, AAT inhibition of *ADCY4* gene expression would be predicted to augment inflammasome activation. |
| ***CREB3L2*** | CAMP responsive element binding protein 3 like 2 | Regulates the secretory capacity of cells by maintaining the volume and structure of the Golgi complex and have been found to modulate nerve growth factor-induced cell differentiation (71, 72). |
| ***IRS1*** | Insulin Receptor Substrate 1 (IRS1) | IRS-1 transmits signals from the insulin receptor and insulin-like growth factor receptor to downstream signaling pathways (73). TNF inhibits the phosphorylation of IRS-1 and thus inhibits response to insulin (74). |
| ***MAPK8*** | Mitogen-Activated Protein Kinase 8 | This protein phosphorylates substrates that increase apoptosis, including p53 and Bcl-2 modulating factor (75). During *MTB* infection, this protein is dephosphorylated, inhibiting the production of various cytokines such as TNF and IL1β (76). |
| ***PIK3R1*** | Phosphoinositide-3-kinase, regulatory subunit 1 (PIK3R1, aka p85α) | p85α, a regulatory subunit of phosphoinositide 3-kinases (PI3K), stabilizes the p110 catalytic subunit and inhibits the latter’s lipid kinase activity (77, 78). p85α functions as a tumor suppressor (79) and plays a critical role in insulin signaling and c-Jun N-terminal kinase-mediated insulin resistance (80). p85α also inhibits the p110δ subunit, which plays an important role in the production of macrophages, an important regulator of *MTB* infection (78, 81) |
| **PRKACB** | Protein kinase A catalytic subunit Cβ (PRKACβ) | PRKACβ is one of two catalytic subunit of protein kinase A (along with two regulatory subunits) that is induced by cAMP. PRKACβ dampens lipopolysaccharide-induced systemic inflammation (82). May have anti-tumor effect (83). |
| ***PTK2*** | Protein Tyrosine Kinase 2, aka FAK (focal adhesion kinase) | During *MTB* infection of macrophages, FAK blocks necrotic cell death and induces ROS production, both of which restrict the survival of *MTB* (84). |
| ***SHC2*** | SHC Adaptor Protein 2 | The association of phospholipase C gamma 2 with the Shc adapter protein (with subsequent activation of p38^mapk^ pathway) mediates the production of reactive oxygen intermediates in *MTB*-infected neutrophils (85). |
| ***SOS2*** | Son of sevenless homolog 2 | SOS2 (known as a guanine nucleotide exchange factor), binds to RAS, causing the latter to release GDP and bind instead GTP, creating the active RAS-GTPases, which hydrolyzes GTP to GDP and phosphate. Thus, SOS2 causes activation of both RAS and downstream kinases and may play a role in the pathogenesis of certain cancer (86). |

*Preferential focus on host-defense function against mycobacterial (microbial) pathogens

**Supplemental Table 9. AAT-inhibited, GR-dependent TGFβ signaling genes**

| **Gene** | **Protein** | **Selected key function*** |
| --- | --- | --- |
| **BMP4** | Bone Morphogenetic Protein 4 | A secreted ligand of the TGFβ superfamily. BMP4 downregulates both IFNγ-producing CD4^+^ T cells and HIF1α expression (87). |
| **EP300** | p300 | A histone acetyltransferase that promotes transcription by reducing the interaction between histone proteins and DNA. Wildtype p300 prevents cancer development whereas mutated p300 may be oncogenic. CREB-binding protein/p300 co-activator plays an important role in *MTB*-induced TNF expression in monocytes (88). |
| **ID1** | DNA-binding protein inhibitor (Id-1) | Id-1 is essential for the differentiation of T_H_9 cells (89). T_H_9 cells impair T_H_1 response in TB (90) as well as induce differentiation of T_H_17 cells and enhance function of regulatory T cells (91). |
| **ID3** | DNA-binding protein inhibitor (Id-3) | Id-3 promotes the development of memory CD8^+^ T cells (92). Id-3 appears to have a dual and opposing roles in germinal B cell evolution: it is essential for germinal center B cell maturation (specification) but is down-regulated at latter stages to promote B cell differentiation to plasma cells (93). |
| **INHBA** | Inhibin subunit beta A protein | This protein activates the TGFβ pathway, leading to proliferation and migration of cancer cells (94). TGFβ is generally considered to predispose to an active *MTB* infection (95) but in conjunction with IL-23, promotes the expansion of T_H_17 cells during mycobacterial infection (96). |
| **RPS6KB2** | Ribosomal Protein S6 Kinase B2 (S6K2) | The primary function of S6K2 is to regulate the cell cycle, but it has also been found to enhance T cell differentiation (97). Along with S6K1, this protein phosphorylates IRF7, stimulating production of type 1 IFN, an important player in *MTB* infection (98). |
| **SMAD2** | Suppressor of Mothers against Decapentaplegic 2 (Smad2) | Smad2 is a downstream transcriptional activator of TGFβ signaling. Critical tumor suppressor and mediator of TGFβ responses in epithelial cells (99). Essential for TGFβ−mediated induction of T regulatory cells and suppression of IFNγ CD4^+^ T cells (100). |
| **SMAD9** | Suppressor of Mothers against Decapentaplegic 9 (Smad9) | Smad9 expression is increased by bone morphogenic protein but Smad9 also inhibits downstream signaling event of BMP, inhibiting BMP-induced transcription (a form of negative feedback) (101). |

*Preferential focus on host-defense function against mycobacterial (microbial) pathogens

**References**

1. **Romero-Adrian TB, Leal-Montiel J, Fernández G, Valecillo A**. Role of cytokines and other factors involved in the *Mycobacterium tuberculosis* infection. *World J Immunol*; 5: 16-50, 2015.

2. **Ratnam NM, Peterson JM, Talbert EE, Ladner KJ, Rajasekera PV, Schmidt CR, Dillhoff ME, Swanson BJ, Haverick E, Kladney RD, Williams TM, Leone GW, Wang DJ, Guttridge DC**. NF-κB regulates GDF-15 to suppress macrophage surveillance during early tumor development. *J Clin Invest*; 127: 3796-3809, 2017.

3. **Majhi RK, Mohanty S, Kamolvit W, White JK, Scheffschick A, Brauner H, Brauner A**. Metformin strengthens uroepithelial immunity against *E. coli* infection. *Sci Rep*; 11: 19263, 2021.

4. **Reyes J, Yap GS**. Emerging Roles of Growth Differentiation Factor 15 in Immunoregulation and Pathogenesis. *J Immunol*; 210: 5-11, 2023.

5. **Huber AK, Finkelman FD, Li CW, Concepcion E, Smith E, Jacobson E, Latif R, Keddache M, Zhang W, Tomer Y**. Genetically driven target tissue overexpression of CD40: a novel mechanism in autoimmune disease. *J Immunol*; 189: 3043-3053, 2012.

6. **Klug-Micu GM, Stenger S, Sommer A, Liu PT, Krutzik SR, Modlin RL, Fabri M**. CD40 ligand and interferon-γ induce an antimicrobial response against *Mycobacterium tuberculosis* in human monocytes. *Immunology*; 139: 121-128, 2013.

7. **Enriquez AB, Sia JK, Dkhar HK, Goh SL, Quezada M, Stallings KL, Rengarajan J**. *Mycobacterium tuberculosis* impedes CD40-dependent notch signaling to restrict Th17 polarization during infection. *iScience*; 25: 104305, 2022.

8. **Chinnam M, Xu C, Lama R, Zhang X, Cedeno CD, Wang Y, Stablewski AB, Goodrich DW, Wang X**. MDM2 E3 ligase activity is essential for p53 regulation and cell cycle integrity. *PLoS Genet*; 18: e1010171, 2022.

9. **Chinnam M, Xu C, Lama R, Zhang X, Cedeno CD, Wang Y, Stablewski AB, Goodrich DW, Wang X**. Correction: MDM2 E3 ligase activity is essential for p53 regulation and cell cycle integrity. *PLoS Genet*; 18: e1010293, 2022.

10. **Lim YJ, Lee J, Choi JA, Cho SN, Son SH, Kwon SJ, Son JW, Song CH**. M1 macrophage dependent-p53 regulates the intracellular survival of mycobacteria. *Apoptosis*; 25: 42-55, 2020.

11. **Ortiz LA, Dutreil M, Fattman C, Pandey AC, Torres G, Go K, Phinney DG**. Interleukin 1 receptor antagonist mediates the antiinflammatory and antifibrotic effect of mesenchymal stem cells during lung injury. *Proc Natl Acad Sci U S A*; 104: 11002-11007, 2007.

12. **Mayer-Barber KD, Andrade BB, Oland SD, Amaral EP, Barber DL, Gonzales J, Derrick SC, Shi R, Kumar NP, Wei W, Yuan X, Zhang G, Cai Y, Babu S, Catalfamo M, Salazar AM, Via LE, Barry CE, 3rd., Sher A**. Host-directed therapy of tuberculosis based on interleukin-1 and type I interferon crosstalk. *Nature*; 511: 99-103, 2014.

13. **Pecce V, Verrienti A, Fiscon G, Sponziello M, Conte F, Abballe L, Durante C, Farina L, Filetti S, Paci P**. The role of FOSL1 in stem-like cell reprogramming processes. *Sci Rep*; 11: 14677, 2021.

14. **Shetty A, Tripathi SK, Junttila S, Buchacher T, Biradar R, Bhosale SD, Envall T, Laiho A, Moulder R, Rasool O, Galande S, Elo LL, Lahesmaa R**. A systematic comparison of FOSL1, FOSL2 and BATF-mediated transcriptional regulation during early human Th17 differentiation. *Nucleic Acids Res*; 50: 4938-4958, 2022.

15. **Yu CP, Pan YL, Wang XL, Xin R, Li HQ, Lei YT, Zhao FF, Zhang D, Zhou XR, Ma WW, Wang SY, Wu YH**. Stimulating the expression of sphingosine kinase 1 (SphK1) is beneficial to reduce acrylamide-induced nerve cell damage. *Ecotoxicol Environ Saf*; 237: 113511, 2022.

16. **Prakash H, Lüth A, Grinkina N, Holzer D, Wadgaonkar R, Gonzalez AP, Anes E, Kleuser B**. Sphingosine kinase-1 (SphK-1) regulates *Mycobacterium smegmatis* infection in macrophages. . *PLoS One*; 5: e10657, 2010.

17. **Ramanadham S, Ali T, Ashley JW, Bone RN, Hancock WD, Lei X**. Calcium-independent phospholipases A2 and their roles in biological processes and diseases. *J Lipid Res*; 56: 1643-1668, 2015.

18. **Duan L, Gan H, Arm J, Remold HG**. Cytosolic phospholipase A2 participates with TNF-alpha in the induction of apoptosis of human macrophages infected with *Mycobacterium tuberculosis* H37Ra. *J Immunol*; 166: 7469-7476, 2001.

19. **Bui TM, Wiesolek HL, Sumagin R**. ICAM-1: A master regulator of cellular responses in inflammation, injury resolution, and tumorigenesis. *J Leukoc Biol*; 108: 787-799, 2020.

20. **Windish HP, Lin PL, Mattila JT, Green AM, Onuoha EO, Kane LP, Flynn JL**. Aberrant TGF-beta signaling reduces T regulatory cells in ICAM-1-deficient mice, increasing the inflammatory response to *Mycobacterium tuberculosis*. *J Leukoc Biol*; 86: 713-725, 2009.

21. **Gilder AS, Natali L, Van Dyk DM, Zalfa C, Banki MA, Pizzo DP, Wang H, Klemke RL, Mantuano E, Gonias SL**. The Urokinase Receptor Induces a Mesenchymal Gene Expression Signature in Glioblastoma Cells and Promotes Tumor Cell Survival in Neurospheres. *Sci Rep*; 8: 2982, 2018.

22. **Rao NK, Shi GP, Chapman HA**. Urokinase receptor is a multifunctional protein: influence of receptor occupancy on macrophage gene expression. *J Clin Invest*; 96: 465-474, 1995.

23. **Gyetko MR, Todd RF, 3rd., Wilkinson CC, Sitrin RG**. The urokinase receptor is required for human monocyte chemotaxis in vitro. *J Clin Invest*; 93: 1380-1387, 1994.

24. **Huang S, Xu M, Liu L, Yang J, Wang H, Wan C, Deng W, Tang Q**. Autophagy is involved in the protective effect of p21 on LPS-induced cardiac dysfunction. *Cell Death Dis*; 11: 554, 2020.

25. **Xaus J, Cardó M, Valledor AF, Soler C, Lloberas J, Celada A**. Interferon gamma induces the expression of p21waf-1 and arrests macrophage cell cycle, preventing induction of apoptosis. *Immunity*; 11: 103-113, 1999.

26. **Silva CA, Ribeiro-Dos-Santos A, Gonçalves WG, Pinto P, Pantoja RP, Vinasco-Sandoval T, Ribeiro-Dos-Santos AM, Hutz MH, Vidal AF, Araújo GS, Ribeiro-Dos-Santos Â, Santos S**. Can miRNA Indicate Risk of Illness after Continuous Exposure to *M. tuberculosis*? *Int J Mol Sci*; 22: 3674, 2021.

27. **Pandey AK, Yang Y, Jiang Z, Fortune SM, Coulombe F, Behr MA, Fitzgerald KA, Sassetti CM, Kelliher MA**. NOD2, RIP2 and IRF5 play a critical role in the type I interferon response to *Mycobacterium tuberculosis*. *PLoS Pathog*; 5: e1000500, 2009.

28. **Morishita D, Katayama R, Sekimizu K, Tsuruo T, Fujita N**. Pim kinases promote cell cycle progression by phosphorylating and down-regulating p27Kip1 at the transcriptional and posttranscriptional levels. *Cancer Res*; 68: 5076-5085, 2008.

29. **Ni J, Zhao J, Zhang X, Reinheckel T, Turk V, Nakanishi H**. Cathepsin H deficiency decreases hypoxia-ischemia-induced hippocampal atrophy in neonatal mice through attenuated TLR3/IFN-β signaling. *J Neuroinflammation*; 18: 176, 2021.

30. **Kamboj RC, Raghav N, Mittal A, Khurana S, Sadana R, Singh H**. Effects of some antituberculous and anti-leprotic drugs on cathepsins B, H and L. *Indian J Clin Biochem*; 18: 39-47, 2003.

31. **Fagerberg L, Hallström BM, Oksvold P, Kampf C, Djureinovic D, Odeberg J, Habuka M, Tahmasebpoor S, Danielsson A, Edlund K, Asplund A, Sjöstedt E, Lundberg E, Szigyarto CA, Skogs M, Takanen JO, Berling H, Tegel H, Mulder J, Nilsson P, Schwenk JM, Lindskog C, Danielsson F, Mardinoglu A, Sivertsson A, von Feilitzen K, Forsberg M, Zwahlen M, Olsson I, Navani S, Huss M, Nielsen J, Ponten F, Uhlén M**. Analysis of the human tissue-specific expression by genome-wide integration of transcriptomics and antibody-based proteomics. *Mol Cell Proteomics*; 13: 397-406, 2014.

32. **Tzelepis F, Blagih J, Khan N, Gillard J, Mendonca L, Roy DG, Ma EH, Joubert P, Jones RG, Divangahi M**. Mitochondrial cyclophilin D regulates T cell metabolic responses and disease tolerance to tuberculosis. *Sci Immunol*; 3: eaar4135, 2018.

33. **Whang J, Back YW, Lee KI, Fujiwara N, Paik S, Choi CH, Park JK, Kim HJ**. *Mycobacterium abscessus* glycopeptidolipids inhibit macrophage apoptosis and bacterial spreading by targeting mitochondrial cyclophilin D. *Cell Death Dis*; 8: e3012, 2017.

34. **Chang T-P, Vancurova I**. Bcl3 regulates pro-survival and pro-inflammatory gene expression in cutaneous T-cell lymphoma. *Biochim Biophys Acta*; 1843: 2620-2630, 2014.

35. **Patel NR, Swan K, Li X, Tachado SD, Koziel H**. Impaired *M. tuberculosis*-mediated apoptosis in alveolar macrophages from HIV+ persons: potential role of IL-10 and BCL-3. *J Leukoc Biol*; 86: 53-60, 2009.

36. **Wu C, Chen Z, Xiao S, Thalhamer T, Madi A, Han T, Kuchroo V**. SGK1 Governs the Reciprocal Development of Th17 and Regulatory T Cells. *Cell Rep*; 22: 653-665, 2018.

37. **Li C, Wei J, Li Y, He X, Zhou Q, Yan J, Zhang J, Liu Y, Liu Y, Shu HB**. Transmembrane Protein 214 (TMEM214) mediates endoplasmic reticulum stress-induced caspase 4 enzyme activation and apoptosis. *J Biol Chem*; 288: 17908-17917, 2013.

38. **Seimon TA, Kim MJ, Blumenthal A, Koo J, Ehrt S, Wainwright H, Bekker LG, Kaplan G, Nathan C, Tabas I, Russell DG**. Induction of ER stress in macrophages of tuberculosis granulomas. *PLoS One*; 5: e12772, 2010.

39. **Kumar NP, Moideen K, Nancy A, Viswanathan V, Thiruvengadam K, Sivakumar S, Hissar S, Nair D, Banurekha VV, Kornfeld H, Babu S**. Association of Plasma Matrix Metalloproteinase and Tissue Inhibitors of Matrix Metalloproteinase Levels With Adverse Treatment Outcomes Among Patients With Pulmonary Tuberculosis. *JAMA Netw Open*; 3: e2027754, 2020.

40. **Liu QY, Han F, Pan LP, Jia HY, Li Q, Zhang ZD**. Inflammation responses in patients with pulmonary tuberculosis in an intensive care unit. *Exp Ther Med*; 15: 2719-2726, 2018.

41. **Shi M, Zhang Y, Liu L, Zhang T, Han F, Cleveland J, Wang F, McKeehan WL, Li Y, Zhang D**. MAP1S Protein Regulates the Phagocytosis of Bacteria and Toll-like Receptor (TLR) Signaling. *J Biol Chem*; 291: 1243-1250, 2016.

42. **Liu L, McKeehan WL, Wang F, Xie R**. MAP1S enhances autophagy to suppress tumorigenesis. *Autophagy*; 8: 278-280, 2012.

43. **Lee YC, Jogie-Brahim S, Lee DY, Han J, Harada A, Murphy LJ, Oh Y**. Insulin-like growth factor-binding protein-3 (IGFBP-3) blocks the effects of asthma by negatively regulating NF-κB signaling through IGFBP-3R-mediated activation of caspases. *J Biol Chem*; 286: 17898-17909, 2011.

44. **Bai X, Feldman NE, Chmura K, Ovrutsky AR, Su W-L, Griffin L, Pyeon D, McGibney MT, Strand MJ, Numata M, Murakami S, Gaido L, Honda JR, Kinney WH, Oberley-Deegan RE, Voelker DR, Ordway DJ, Chan ED**. Inhibition of nuclear factor-kappa B activation decreases survival of *Mycobacterium tuberculosis* in human macrophages. *PLoS One*; 8: e61925, 2013.

45. **Otterbein LE, Bach FH, Alam J, Soares M, Tao LH, Wysk M, Davis RJ, Flavell RA, Choi AM**. Carbon monoxide has anti-inflammatory effects involving the mitogen-activated protein kinase pathway. *Nat Med*; 6: 422-428, 2000.

46. **Singh N, Ahmad Z, Baid N, Kumar A**. Host heme oxygenase-1: Friend or foe in tackling pathogens? *IUBMB Life*; 70: 869-880, 2018.

47. **Verhagen AM, Ekert PG, Pakusch M, Silke J, Connolly LM, Reid GE, Moritz RL, Simpson RJ, Vaux DL**. Identification of DIABLO, a mammalian protein that promotes apoptosis by binding to and antagonizing IAP proteins. *Cell*; 102: 43-53, 2000.

48. **Mukaida N, Wang YY, Li YY**. Roles of Pim-3, a novel survival kinase, in tumorigenesis. *Cancer Sci*; 102: 1437-1442, 2011.

49. **Buchacher T, Shetty A, Koskela SA, Smolander J, Kaukonen R, Sousa AGG, Junttila S, Laiho A, Rundquist O, Lönnberg T, Marson A, Rasool O, Elo LL, Lahesmaa R**. PIM kinases regulate early human Th17 cell differentiation. *Cell Rep*; 42: 113469, 2023.

50. **Wang Y, Han E, Xing Q, Yan J, Arrington A, Wang C, Tully D, Kowolik CM, Lu DM, Frankel PH, Zhai J, Wen W, Horne D, Yip MLR, Yim JH**. Baicalein upregulates DDIT4 expression which mediates mTOR inhibition and growth inhibition in cancer cells. *Cancer Lett*; 358: 170-179, 2015.

51. **Shoshani T, Faerman A, Mett I, Zelin E, Tenne T, Gorodin S, Moshel Y, Elbaz S, Budanov A, Chajut A, Kalinski H, Kamer I, Rozen A, Mor O, Keshet E, Leshkowitz D, Einat P, Skaliter R, Feinstein E**. Identification of a novel hypoxia-inducible factor 1-responsive gene, RTP801, involved in apoptosis. *Mol Cell Biol*; 22: 2283-2293, 2002.

52. **Liang J, Wang J, Azfer A, Song W, Tromp G, Kolattukudy PE, Fu M**. A novel CCCH-zinc finger protein family regulates proinflammatory activation of macrophages. *J Biol Chem*; 283: 6337–6346, 2008.

53. **Lu L, Wei R, Bhakta S, Waddell SJ, Boix E**. Weighted Gene Co-Expression Network Analysis Identifies Key Modules and Hub Genes Associated with Mycobacterial Infection of Human Macrophages. *Antibiotics (Basel)*; 10: 97, 2021.

54. **Kumar H, Kawai T, Akira S**. Toll-like receptors and innate immunity. *Biochem Biophys Res Commun*; 388: 621-625, 2009.

55. **Boni FG, Hamdi I, Koundi LM, Shrestha K, Xie J**. Cytokine storm in tuberculosis and IL-6 involvement. *Infect Genet Evol*; 97: 105166, 2022.

56. **Vidarsdottir L, Fernandes RV, Zachariadis V, Das I, Edsbäcker E, Sigvaldadottir I, Azimi A, Höiom V, Hansson J, Grandér D, Egyházi Brage S, Pokrovskaja Tamm K**. Silencing of CEBPB-AS1 modulates CEBPB expression and resensitizes BRAF-inhibitor resistant melanoma cells to vemurafenib. *Melanoma Res*; 30: 443-454, 2020.

57. **Sahu SK, Kumar M, Chakraborty S, Banerjee SK, Kumar R, Gupta P, Jana K, Gupta UD, Ghosh Z, Kundu M, Basu J**. MicroRNA 26a (miR-26a)/KLF4 and CREB-C/EBPβ regulate innate immune signaling, the polarization of macrophages and the trafficking of *Mycobacterium tuberculosis* to lysosomes during infection. *PLoS Pathog*; 13: e1006410, 2017.

58. **Perng YC, Lenschow DJ**. ISG15 in antiviral immunity and beyond. *Nat Rev Microbiol*; 16: 423-439, 2018.

59. **Bustamante J, Boisson-Dupuis S, Abel L, Casanova JL**. Mendelian susceptibility to mycobacterial disease: genetic, immunological, and clinical features of inborn errors of IFN-γ immunity. *Semin Immunol*; 26: 454-470, 2014.

60. **Kimmey JM, Campbell JA, Weiss LA, Monte KJ, Lenschow DJ, Stallings CL**. The impact of ISGylation during *Mycobacterium tuberculosis* infection in mice. *Microbes Infect*; 19: 249-258, 2017.

61. **Xu J-L, Guo Y**. FCGR1A Serves as a Novel Biomarker and Correlates With Immune Infiltration in Four Cancer Types. *Front Mol Biosci*; 7: 581615, 2020.

62. **Jenum S, Bakken R, Dhanasekaran S, Mukherjee A, Lodha R, Singh S, Singh V, Haks MC, Ottenhoff TH, Kabra SK, Doherty TM, Ritz C, Grewal HM**. BLR1 and FCGR1A transcripts in peripheral blood associate with the extent of intrathoracic tuberculosis in children and predict treatment outcome. *Sci Rep*; 6: 38841, 2016.

63. **Sutherland JS, Loxton AG, Haks MC, Kassa D, Ambrose L, Lee JS, Ran L, van Baarle D, Maertzdorf J, Howe R, Mayanja-Kizza H, Boom WH, Thiel BA, Crampin AC, Hanekom W, Ota MO, Dockrell H, Walzl G, Kaufmann SH, Ottenhoff TH**. GCGH Biomarkers for TB consortium. Differential gene expression of activating Fcγ receptor classifies active tuberculosis regardless of human immunodeficiency virus status or ethnicity. *Clin Microbiol Infect*; 20: O230-238, 2014.

64. **Sadanari H, Takemoto M, Ishida T, Otagiri H, Daikoku T, Murayama T, Kusano S**. The Interferon-Inducible Human PLSCR1 Protein Is a Restriction Factor of Human Cytomegalovirus. *Microbiol Spectr*; 10: e0134221, 2022.

65. **Ranjbar S, Haridas V, Jasenosky LD, Falvo JV, Goldfeld AE**. A Role for IFITM Proteins in Restriction of *Mycobacterium tuberculosis* Infection. *Cell Rep*; 13: 874-883, 2015.

66. **Ning S, Pagano JS, Barber GN**. IRF7: activation, regulation, modification and function. *Genes Immun*; 12: 399-414, 2011.

67. **Mogensen TH**. IRF and STAT Transcription Factors - From Basic Biology to Roles in Infection, Protective Immunity, and Primary Immunodeficiencies. *Front Immunol*; 9: 3047, 2019.

68. **Zhang Z, Mai Q, Yang L, Chen Y, Chen Z, Lin T, Tan S, Wu Z, Cai Y, Cui T, Ouyang B, Yang Y, Zeng L, Ge Z, Zhang S, Zeng G, Pi J, Chen L**. MicroRNA-31 mediated by interferon regulatory factor 7 signaling facilitates control of *Mycobacterium tuberculosis* infection. . *Int J Med Microbiol*; 312: 151569, 2022.

69. **Fan Y, Mu J, Huang M, Imani S, Wang Y, Lin S, Fan J, Wen Q**. Epigenetic identification of ADCY4 as a biomarker for breast cancer: an integrated analysis of adenylate cyclases. *Epigenomics*; 11: 1561-1579, 2019.

70. **Chen R, Zeng L, Zhu S, Liu J, Zeh HJ, Kroemer G, Wang H, Billiar TR, Jiang J, Tang D, Kang R**. cAMP metabolism controls caspase-11 inflammasome activation and pyroptosis in sepsis. *Sci Adv*; 5: eaav5562, 2019.

71. **Sampieri L, Funes Chabán M, Di Giusto P, Rozés-Salvador V, Alvarez C**. CREB3L2 Modulates Nerve Growth Factor-Induced Cell Differentiation. *Front Mol Neurosci*; 14: 650338, 2021.

72. **Pittari D, Dalla Torre M, Borini E, Hummel B, Sawarkar R, Semino C, van Anken E, Panina-Bordignon P, Sitia R, Anelli T**. CREB3L1 and CREB3L2 control Golgi remodelling during decidualization of endometrial stromal cells. *Front Cell Dev Biol*; 10: 986997, 2022.

73. **Boucher J, Kleinridders A, Kahn CR**. Insulin receptor signaling in normal and insulin-resistant states. *Cold Spring Harb Perspect Biol*; 6: a009191, 2014.

74. **Hotamisligil GS, Murray DL, Choy LN, Spiegelman BM**. Tumor necrosis factor alpha inhibits signaling from the insulin receptor. *Proc Natl Acad Sci U S A*; 91: 4854-4858, 1994.

75. **Nikiforov VS, Blinova EA, Kotikova AI, Akleyev AV**. Transcriptional activity of repair, apoptosis and cell cycle genes (TP53, MDM2, ATM, BAX, BCL-2, CDKN1A, OGG1, XPC, PADI4, MAPK8, NF-KB1, STAT3, GATA3) in chronically exposed persons with different intensity of apoptosis of peripheral blood lymphocytes. *Vavilovskii Zhurnal Genet Selektsii*; 26: 50-58, 2022.

76. **Shariq M, Quadir N, Alam A, Zarin S, Sheikh JA, Sharma N, Samal J, Ahmad U, Kumari I, Hasnain SE, Ehtesham NZ**. The exploitation of host autophagy and ubiquitin machinery by *Mycobacterium tuberculosis* in shaping immune responses and host defense during infection. *Autophagy*; 19: 3-23, 2023.

77. **Tsay A, Wang JC**. The Role of PIK3R1 in Metabolic Function and Insulin Sensitivity. *Int J Mol Sci*; 24: 12665, 2023.

78. **Yu J, Zhang Y, McIlroy J, Rordorf-Nikolic T, Orr GA, Backer JM**. Regulation of the p85/p110 phosphatidylinositol 3'-kinase: stabilization and inhibition of the p110alpha catalytic subunit by the p85 regulatory subunit. *Mol Cell Biol*; 18: 1379-1387, 1998.

79. **Vallejo-Díaz J, Chagoyen M, Olazabal-Morán M, González-García A, Carrera AC**. The Opposing Roles of PIK3R1/p85α and PIK3R2/p85β in Cancer. *Trends Cancer*; 5: 233-244, 2019.

80. **Taniguchi CM, Aleman JO, Ueki K, Luo J, Asano T, Kaneto H, Stephanopoulos G, Cantley LC, Kahn CR**. The p85alpha regulatory subunit of phosphoinositide 3-kinase potentiates c-Jun N-terminal kinase-mediated insulin resistance. *Mol Cell Biol*; 27: 2830-2840, 2007.

81. **Yu CH, Micaroni M, Puyskens A, Schultz TE, Yeo JC, Stanley AC, Lucas M, Kurihara J, Dobos KM, Stow JL, Blumenthal A**. RP105 Engages Phosphatidylinositol 3-Kinase p110δ To Facilitate the Trafficking and Secretion of Cytokines in Macrophages during Mycobacterial Infection. *J Immunol*; 195: 3890-3900, 2015.

82. **Moen LV, Sener Z, Volchenkov R, Svarstad AC, Eriksen AM, Holen HL, Skålhegg BS**. Ablation of the Cβ2 subunit of PKA in immune cells leads to increased susceptibility to systemic inflammation in mice. *Eur J Immunol*; 47: 1880-1889, 2017.

83. **Yao X, Hu W, Zhang J, Huang C, Zhao H, Yao X**. Application of cAMP-dependent catalytic subunit β (PRKACB) Low Expression in Predicting Worse Overall Survival: A Potential Therapeutic Target for Colorectal Carcinoma. *J Cancer*; 11: 4841-4850, 2020.

84. **Afriyie-Asante A, Dabla A, Dagenais A, Berton S, Smyth R, Sun J**. *Mycobacterium tuberculosis* Exploits Focal Adhesion Kinase to Induce Necrotic Cell Death and Inhibit Reactive Oxygen Species Production. . *Front Immunol*; 12: 742370, 2021.

85. **Perskvist N, Zheng L, Stendahl O**. Activation of human neutrophils by *Mycobacterium tuberculosis* H37Ra involves phospholipase C gamma 2, Shc adapter protein, and p38 mitogen-activated protein kinase. *J Immunol*; 164: 959-965, 2000.

86. **Liceras-Boillos P, Jimeno D, García-Navas R, Lorenzo-Martín LF, Menacho-Marquez M, Segrelles C, Gómez C, Calzada N, Fuentes-Mateos R, Paramio JM, Bustelo XR, Baltanás FC, Santos E**. Differential Role of the RasGEFs Sos1 and Sos2 in Mouse Skin Homeostasis and Carcinogenesis. *Mol Cell Biol*; 38: e00049-00018, 2018.

87. **Huang F, Hu L, Zhang Y, Qu X, Xu J**. BMP4 Moderates Glycolysis and Regulates Activation and Interferon-Gamma Production in CD4+ T Cells. *Front Immunol*; 12: 702211, 2021.

88. **Barthel R, Tsytsykova AV, Barczak AK, Tsai EY, Dascher CC, Brenner MB, Goldfeld AE**. Regulation of tumor necrosis factor alpha gene expression by mycobacteria involves the assembly of a unique enhanceosome dependent on the coactivator proteins CBP/p300. *Mol Cell Biol*; 23: 526-533, 2003.

89. **Lee WH, Hong KJ, Li HB, Lee GR**. Transcription Factor Id1 Plays an Essential Role in Th9 Cell Differentiation by Inhibiting Tcf3 and Tcf4. *Adv Sci (Weinh)*; 10: e2305527, 2023.

90. **Wu B, Huang C, Kato-Maeda M, Hopewell PC, Daley CL, Krensky AM, Clayberger C**. IL-9 is associated with an impaired Th1 immune response in patients with tuberculosis. *Clin Immunol*; 126: 202-210, 2008.

91. **Elyaman W, Bradshaw EM, Uyttenhove C, Dardalhon V, Awasthi A, Imitola J, Bettelli E, Oukka M, van Snick J, Renauld JC, Kuchroo VK, Khoury SJ**. IL-9 induces differentiation of TH17 cells and enhances function of FoxP3+ natural regulatory T cells. *Proc Natl Acad Sci U S A*; 106: 12885-12890, 2009.

92. **Hu G, Chen J**. A genome-wide regulatory network identifies key transcription factors for memory CD8⁺ T-cell development. *Nat Commun*; 4: 2830, 2013.

93. **Chen S, Miyazaki M, Chandra V, Fisch KM, Chang AN, Murre C**. Id3 Orchestrates Germinal Center B Cell Development. *Mol Cell Biol*; 36: 2543-2552, 2016.

94. **Zhang H, Huang Y, Wen Q, Li Y, Guo L, Ge N**. INHBA gene silencing inhibits proliferation, migration, and invasion of osteosarcoma cells by repressing TGF-β signaling pathway activation. . *J Orthop Surg Res*; 18: 848, 2023.

95. **Gern BH, Adams KN, Plumlee CR, Stoltzfus CR, Shehata L, Moguche AO, Busman-Sahay K, Hansen SG, Axthelm MK, Picker LJ, Estes JD, Urdahl KB, Gerner MY**. TGFβ restricts expansion, survival, and function of T cells within the tuberculous granuloma. *Cell Host Microbe*; 29: 594-606, 2021.

96. **Basile JI, Kviatcovsky D, Romero MM, Balboa L, Monteserin J, Ritacco V, Lopez B, Sabio y García C, García A, Vescovo M, Montaner PG, Palmero D, Del Carmen Sasiain M, de la Barrera S**. *Mycobacterium tuberculosi*s multi-drug-resistant strain M induces IL-17+ IFNγ- CD4+ T cell expansion through an IL-23 and TGF-β-dependent mechanism in patients with MDR-TB tuberculosis. *Clin Exp Immunol*; 187: 160-173, 2017.

97. **Kurebayashi Y, Nagai S, Ikejiri A, Ohtani M, Ichiyama K, Baba Y, Yamada T, Egami S, Hoshii T, Hirao A, Matsuda S, Koyasu S**. PI3K-Akt-mTORC1-S6K1/2 axis controls Th17 differentiation by regulating Gfi1 expression and nuclear translocation of RORγ. *Cell Rep*; 1: 360-373, 2012.

98. **Weichhart T, Hengstschläger M, Linke M**. Regulation of innate immune cell function by mTOR. *Nat Rev Immunol*; 15: 599-614, 2015.

99. **Yang J, Wahdan-Alaswad R, Danielpour D**. Critical role of Smad2 in tumor suppression and transforming growth factor-beta-induced apoptosis of prostate epithelial cells. *Cancer Res*; 69: 2185-2190, 2009.

100. **Takimoto T, Wakabayashi Y, Sekiya T, Inoue N, Morita R, Ichiyama K, Takahashi R, Asakawa M, Muto G, Mori T, Hasegawa E, Saika S, Hara T, Nomura M, Yoshimura A**. Smad2 and Smad3 are redundantly essential for the TGF-beta-mediated regulation of regulatory T plasticity and Th1 development. *J Immunol*; 185: 842-855, 2010.

101. **Tsukamoto S, Mizuta T, Fujimoto M, Ohte S, Osawa K, Miyamoto A, Yoneyama K, Murata E, Machiya A, Jimi E, Kokabu S, Katagiri T**. Smad9 is a new type of transcriptional regulator in bone morphogenetic protein signaling. *Sci Rep*; 4: 7596, 2014.
